# Niacin promotes motor function recovery after spinal cord injury via Hcar2‐dependent microglia immunometabolic regulation

**DOI:** 10.1002/ctm2.70683

**Published:** 2026-05-01

**Authors:** Hua Du, Lingnian Zeng, Chan Liu, Huyao Zhou, Xia Wang, Qing Ai, Jinpiao Zhu, Nong Xiao

**Affiliations:** ^1^ Department of Rehabilitation Children's Hospital of Chongqing Medical University, National Clinical Research Center for Children and Adolescents’ Health and Diseases, Ministry of Education Key Laboratory of Child Development and Disorders, Chongqing Key Laboratory of Child Neurodevelopment and Cognitive Disorders Chongqing China; ^2^ Perioperative and Systems Medicine Laboratory Department of Rehabilitation Children's Hospital Zhejiang University School of Medicine, National Clinical Research Center for Children and Adolescent's Health and Diseases Hangzhou China

**Keywords:** hydroxycarboxylic acid receptor 2, immunometabolism, metabolic reprogramming, microglia, neuroinflammation, niacin, spinal cord injury

## Abstract

**Background:**

Traumatic spinal cord injury (SCI) induces a robust local inflammatory response that can both facilitate repair and exacerbate pathology. Hydroxycarboxylic acid receptor 2 (Hcar2) is known to exert immunomodulatory effects; however, its role in SCI and its potential for targeting Hcar2 to alleviate motor deficits remain unclear.

**Methods:**

The spinal cord transcriptome following SCI, with a focus on *Hcar2*, was analysed via publicly available single‐cell RNA sequencing datasets from mice and rhesus macaques. Additionally, an in vivo SCI mouse model with *Hcar2* knockout and an in vitro LPS‐induced BV2 microglial model were established to assess *Hcar2* gene and protein expression, microglial activation and inflammatory responses via bulk RNA sequencing, immunofluorescence staining, Western blotting, and real‐time polymerase chain reaction. To evaluate the protective effects of Hcar2 activation, niacin, a known Hcar2 agonist, was administered to mice or BV2 cells, followed by assessments of the inflammatory response and motor function.

**Results:**

*Hcar2* gene expression, which was enriched predominantly in spinal cord microglia, was upregulated following SCl, peaking at 7 days post‐SCl. Genetic knockout of *Hcar2* decreased the percentage of impaired anti‐inflammatory polarized microglia and increased the inflammatory response. In contrast, Hcar2 activation with niacin in LPS‐stimulated microglia BV cell models reversed mitochondrial dysfunction, increased the oxygen consumption rate and reduced the expression of the cytokines *IL‐6* and *IL‐1β*. The administration of niacin to SCl mice upregulated anti‐inflammatory microglia, reduced the expression of multiple proinflammatory cytokines, increased the number of motor neurons and improved motor function recovery. Notably, all these protective effects were abolished by genetic loss of *Hcar2*.

**Conclusions:**

Hcar2 serves as a critical regulator of microglial polarization, promoting the switch from a proinflammatory phenotype to an anti‐inflammatory phenotype through immunometabolic reprogramming. Targeting Hcar2 with niacin may offer a translatable therapeutic strategy to improve functional recovery after SCl.

**Key Points:**

- Hcar2 is identified as a conserved, injury‐induced metabolic checkpoint specifically enriched in microglia following spinal cord injury.
- Hcar2 activation reprogrammes microglial metabolism from glycolysis to oxidative phosphorylation to drive reparative anti‐inflammatory polarization.
- Pharmacological targeting of Hcar2 with niacin resolves neuroinflammation and promotes functional motor recovery in an Hcar2‐dependent manner.

## Graphical abstract

• The activation of the microglial Hcar2 receptor by niacin initiates intracellular signalling that induces metabolic reprogramming.

• This metabolic shift is characterized by increased oxidative phosphorylation. Consequently, microglia polarize from a proinflammatory state toward an anti‐inflammatory, reparative phenotype.

• This phenotypic switch attenuates neuroinflammation and neuronal death, fostering a microenvironment favourable for neuroprotection and functional recovery following SCI.

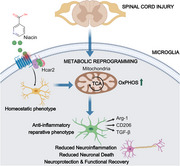

## 1 INTRODUCTION

Traumatic spinal cord injury (SCI) is a devastating neurological condition that leads to long‐term functional deficits and currently lacks effective restorative therapies.^1^, ^2^ In addition to initial mechanical trauma, a prolonged cascade of secondary injury ensues, exacerbating tissue damage and functional loss.^3^ A feature of this secondary pathology is a persistent and dysregulated neuroinflammatory response, which represents a major barrier to endogenous repair and neuronal axonal regeneration.^4^ Therefore, modulating posttraumatic neuroinflammation may serve as a critical therapeutic strategy to mitigate secondary degeneration and promote functional recovery.

This neuroinflammatory milieu of the injured spinal cord is governed primarily by resident microglia and infiltrating macrophages, which are the key immune effectors in the injured central nervous system (CNS).^5^ These myeloid cells show remarkable plasticity and are capable of adopting a proinflammatory (M1‐like) phenotype that exacerbates tissue damage or an anti‐inflammatory (M2‐like) phenotype that facilitates neural repair and regeneration.^6^ Immunometabolic reprogramming is a critical determinant of this phenotypic switch. A metabolic shift toward aerobic glycolysis supports the M1‐like state, whereas reliance on oxidative phosphorylation (OXPHOS) sustains M2‐like reparative functions.^7^ Given that the posttraumatic microenvironment predominantly favours a chronic and neurotoxic M1‐like response,^8^ uncovering endogenous mechanisms that can reprogram microglial metabolism toward an anti‐inflammatory state represents a promising therapeutic strategy after SCI.

Hydroxycarboxylic acid receptor 2 (Hcar2), also known as GPR109A, is a G protein‐coupled receptor that is increasingly recognized for its potent immunomodulatory properties.^9^, ^10^ In the CNS, the activation of Hcar2 by its endogenous ligands, for example, ketone bodies, or pharmacological agonists, for example, niacin,^11^ attenuates microglial activation, suppresses proinflammatory cytokine release, and confers neuroprotection in models of neurodegeneration and chronic pain.^12^, ^13^ Despite its well‐established role in the intersection of metabolism and immune function, the contribution of Hcar2 to the pathophysiology of traumatic SCI has not yet been elucidated.

In the present study, we hypothesized that Hcar2 serves as a critical regulator of microglial metabolic and phenotypic reprogramming following SCI and that targeting Hcar2 could attenuate neuroinflammation and promote motor recovery. We found that *Hcar2* is a microglia‐enriched gene whose expression is upregulated after SCI. Increased Hcar2 expression was essential for driving an anti‐inflammatory microglial phenotype. Moreover, administration of the Hcar2 agonist niacin reduced neuroinflammation and enhanced motor recovery, identifying Hcar2 as a promising and translatable therapeutic target for spinal cord repair.

## 2 MATERIALS AND METHODS

### 2.1 Animals

Female C57BL/6J wild‐type (*Hcar2* ^+/+^) mice (8–10 weeks old, 17–20 g) were obtained from Hunan SJA Laboratory Animal Co., Ltd. *Hcar2* knockout (*Hcar2^−/−^*) mice (Catalog No. NM‐KO‐2114676) were purchased from the Shanghai Model Organisms Center and subsequently bred in‐house. Genotyping was performed using allele‐specific polymerase chain reaction (PCR) primers targeting both the wild‐type and knockout alleles. All mice were housed in a specific pathogen‐free (SPF) facility with ad libitum access to food and water.

### 2.2 SCI Model and Drug Administration

The mice were anaesthetized with 1.5% isoflurane (R510‐22‐10; RWD) and underwent T9 laminectomy. Moderate contusion injury (80 kdyn, 1‐mm tip) was induced via a spinal cord impactor (ZS‐FD/NL; Zhongshi Dichuang Technology Development Co., Ltd.). Sham controls underwent a laminectomy only. Afterwards, the mice were allowed to recover in a controlled chamber, subjected to manual bladder expression three times per day, and given chloramphenicol (50 mg/kg; HY‐B0239; MCE, USA) in their drinking water. For treatment, niacin (NA; Catalog No. N0761‐100G; Sigma‒Aldrich) was administered in the drinking water at a concentration of 3 mg/mL (6 mL/day) for 28 days post‐SCI, and the control groups received sterile water. The in vivo niacin dosage was chosen based on recent preclinical studies confirming its efficacy, CNS penetration and safety in modulating microglial responses in mouse models of neuroinflammation.^14^, ^15^

### 2.3 Behavioural test

Prior to the initiation of behavioural assays, all mice were habituated to the experimental setting for a minimum of 3 days.

#### 2.3.1 CatWalk gait analysis

To evaluate walking coordination and other gait parameters, we performed automated quantitative gait analysis via the CatWalk XT system (Noldus Information Technology) at 28 days post‐injury. The mice were allowed to traverse a glass walkway, and at least three valid runs were recorded for each animal.

#### 2.3.2 Basso Mouse Scale (BMS) scoring

Post‐injury locomotor recovery was quantified using the Mouse Scale (BMS), which systematically evaluates trunk stability, forelimb‐hindlimb coordination, stepping patterns, paw placement, and general hindlimb joint movement. Within this grading framework, a score of 0 defines absolute hindlimb paralysis, while a 9 denotes fully normal locomotion. Open‐field testing sessions, each lasting 4 min, were conducted at 1, 3, 7, 14, 21, and 28 days following SCI. To preclude observational bias, the definitive BMS score for every individual was derived from the mean of independent assessments recorded by two blinded examiners.

### 2.4 Tissue sampling

Under 2% isoflurane anaesthesia, the animals underwent transcardial perfusion using cold phosphate‐buffered saline (PBS; BL601A; Biosharp) followed by 4% paraformaldehyde (PFA; G1101; Servicebio). A 0.5‐cm spinal cord segment encompassing the lesion epicenter was excised and subjected to overnight post‐fixation in 4% PFA at 4°C. For cryoprotection, the specimens were dehydrated sequentially through 18%, 24%, and 30% sucrose gradients at 4°C until complete submersion in the final concentration was achieved. Ultimately, the samples were embedded in optimal cutting temperature compound (Sakura Finetek, Torrance, CA, USA) and flash‐frozen. Cryosectioning of the frozen specimens was performed at a 10‐µm thickness using a CM1950 cryostat microtome (Leica Biosystems), after which the sections were preserved at ‐80°C.

### 2.5 Immunofluorescence staining

Spinal cord sections or cells cultured on coverslips were washed with PBS and fixed with 4% PFA for 10 min(tissue) or 15 min (cells). After permeabilization in PBS containing 0.3% Triton X‐100 (P0096; Beyotime) for 10 min and blocking with 5% BSA in PBS for 1 h at room temperature, samples were incubated overnight at 4°C with the following primary antibodies: rabbit anti‐Hcar2 (1:200; AWA45885; Abiowell), guinea pig anti‐Iba‐1 (1:1000; OB‐PGP049‐01; Oasis), rabbit anti‐Iba‐1 (1:1000; OB‐PGP029‐01; Oasis), mouse anti‐Arg‐1 (1:200; sc‐271430), rabbit anti‐HK2 (1:100; F23A5; Selleck), goat anti‐ChAT (1:100; AWA45885; Abiowell), and chicken anti‐GFAP (1:1000; ab4674; Abcam). After three washes with PBS, the cells or tissue sections were incubated with the corresponding Alexa Fluor‐conjugated secondary antibodies for 1 h at room temperature, protected from light: donkey anti‐guinea pig IgG AF647 (1:1000; D‐GP647; Oasis), goat anti‐rabbit IgG AF568 (1:1000; ab175471; Abcam), goat anti‐rabbit IgG AF488 (1:1000; ab150077; Abcam), goat anti‐mouse IgG AF568 (1:1000; ab175473; Abcam), and goat anti‐chicken IgY AF488 (1:1000; ab150169; Abcam). Nuclei were counterstained with DAPI (C1006, Beyotime) for 5 min. Finally, the slides were mounted with an anti‐fade mounting medium. Images were captured via a confocal laser scanning microscope (CSU‐W1 SoRa; Nikon).

For quantitative analysis, at least three nonoverlapping fields per section were analysed via ImageJ software (v1.54f; NIH). Cell counting and fluorescence intensity quantification were performed by an investigator blinded to the group allocation.

### 2.6 Western blot analysis

Spinal cord tissues or cultured cells were lysed in RIPA buffer (P0013C; Beyotime) supplemented with a protease and phosphatase inhibitor cocktail (GRF103; Epizyme Biotech). Total protein was quantified using a BCA assay kit (ZJ101; Epizyme Biotech). Subsequently, 20 µg protein samples were separated by SDS‐PAGE and electrotransferred onto PVDF membranes (WJ002; Epizyme Biotech). After blocking with 5% nonfat milk, the membranes were incubated overnight at 4°C with the indicated primary antibodies: against Hcar2 (1:2000; AWA45885; Abiowell), Arg‐1 (1:2000; sc‐271430; Santa Cruz Biotechnology), CD206 (1:2000; ET1702‐04; HUABIO), TGF‐β (1:2000; AWA10316; Abiowell), or HRP‐conjugated β‐actin (1:10000; 700068; Zenbio). After a 1‐h incubation at room temperature with the respective HRP‐conjugated secondary antibodies (ab6721/ab205719; Abcam), the immunoreactive signals were developed using an enhanced chemiluminescence (ECL) reagent (P2100; NCM Biotech). Final image acquisition was performed on an iBright™ CL1500 instrument (Invitrogen). Band densities were quantified via ImageJ and normalized to that of β‐actin.

### 2.7 Multiplex flow cytometric analysis

To quantify the levels of IL‐6, IL‐1β and TNF‐α within the spinal cord tissue lysates, a multiplex bead‐based immunoassay kit (XMplex Mouse 4‐Plex Custom Panel; XMPlex01240648; SaiXiaoMan Biotechnology) was employed in strict adherence to the provider's instructions. In brief, individual samples and reference standards were mixed with 5 µL of capture antibody‐coupled beads and maintained at 37°C for 60 min. Following a standard wash step, 50 µL of biotinylated detection antibodies were introduced into the mixture, which then underwent a secondary incubation at 37°C for 30 min. Following another wash, streptavidin‐phycoerythrin (SA‐PE) solution (50 µL) was added, and the reaction mixture underwent a 15‐min incubation at 37°C while protected from light. After a final wash, the beads were resuspended in wash buffer (55 µL). Data were acquired on an ABplex‐100 system (ABclonal), and cytokine concentrations were determined via a standard curve with a four‐parameter logistic fit.

### 2.8 Cell cultures

Murine BV2 microglial cells (PC‐H2025061124; Procell) were propagated in Dulbecco's modified Eagle's medium (DMEM; 11965092; Gibco) enriched with 10% foetal bovine serum (FBS; A5670701; Gibco) and 1% penicillin‐streptomycin (PB180120; Procella) under standard incubation conditions (37°C, 5% CO_2_, humidified atmosphere). Prior to experimental interventions, the cells were plated into 24‐well microplates. Upon reaching 70% to 80% confluence, the cultures were subjected to lipopolysaccharide stimulation (LPS; 100 ng/mL; *Escherichia coli* O111:B4; L2630; Sigma‒Aldrich) with or without cotreatment with niacin (0.1, 0.3, or 1 mM) for 24 h before being harvested for subsequent experiments. The in vitro concentrations were selected in accordance with validated microglial culture protocols to achieve optimal Hcar2 activation without compromising cell viability.^14^

### 2.9 Lentiviral transduction

To establish *Hcar2*‐knockdown microglial cell lines, lentiviral vectors expressing short hairpin RNA (shRNA) targeting mouse *Hcar2* (sh*Hcar2*) and a scrambled negative control (NC) were constructed and packaged by Obio Technology. BV2 cells were seeded in 6‐well plates and infected with the respective lentiviruses at a 20 multiplicity of infection (MOI) in the presence of 1 mg/mL polybrene (PTSJ—GR001; Obio Technology). After 48 h of infection, the cells were selected with puromycin (2 µg/mL) to generate stable knockdown cell lines. The knockdown efficiency was validated by qRT‒PCR prior to subsequent LPS and niacin treatments.

### 2.10 Quantitative reverse transcription polymerase chain reaction (qRT‒PCR)

Total RNA isolation from both spinal cord tissues and BV2 cells was executed using the SteadyPure Quick RNA Extraction Kit (AG21023; Accurate Biology). Following the evaluation of RNA yield and purity via a NanoDrop spectrophotometer (Thermo Fisher Scientific), 1 µg of the isolated RNA per sample was subjected to reverse transcription using the Evo M‐MLV RT Premix Kit (AG11734; Accurate Biology). RT‐qPCR was executed using the SYBR Green Pro Taq HS Premix (AG11733; Accurate Biology) on a CFX96 instrument (Bio‐Rad). The thermal cycling program consisted of an initial denaturation at 95°C for 30 s, followed by 40 cycles of 95°C for 5 s and 60°C for 30 s. Relative gene expression was quantified using the 2^−ΔΔCt^ method and normalized to β‐actin. All reactions were performed in triplicate. Primer sequences are listed in Table S1.

### 2.11 Cellular metabolic assays

#### 2.11.1 Oxygen consumption rate (OCR) assessment

Following the aspiration of the post‐treatment media, the oxygen consumption rate (OCR) was evaluated using a commercial assay kit (E‐BC‐K068‐M; Elabscience). The microplate was immediately transferred to a BioTek Synergy H1 reader configured to dynamic mode at 37°C. Kinetic fluorescence tracking was subsequently executed using excitation and emission wavelengths of 405 and 675 nm, respectively, with data points captured at 2‐min intervals over a 90‐min span. Subsequently, the oxygen consumption rate (OCR) was quantified by calculating the slope of the generated fluorescence‐time curve.

#### 2.11.2 Mitochondrial membrane potential (ΔΨm)

Mitochondrial membrane potential (ΔΨm) was evaluated using a JC‐1 Assay Kit (MA0338; Meilunbio). Following the incubation of coverslip‐cultured BV2 cells with the JC‐1 probe, fluorescent signals were acquired with a CSU‐W1 SoRa confocal laser scanning microscope (Nikon). The relative ΔΨm was subsequently quantified in ImageJ software (v1.54f; NIH) by computing the intensity ratio of red (J‐aggregates) to green (J‐monomers) fluorescence.

#### 2.11.3 ATP measurement

To quantify intracellular ATP concentrations, a commercial ATP Assay Kit (S0026; Beyotime) was employed in strict accordance with the protocol. Following complete cellular lysis, the resulting homogenates were directly reacted with the designated ATP detection working solution. Luminescent signals were promptly quantified using a BioTek Synergy H1 microplate reader (SH1MF‐SN). The corresponding ATP levels were subsequently standardized against the total protein content of each sample, as evaluated by a standard BCA protein assay.

### 2.12 Bioinformatic analysis of publicly available single‐cell RNA sequencing datasets

This study initially integrated and analysed publicly available single‐cell RNA sequencing (scRNA‐seq) data from the injured spinal cords of mice (GEO: GSE162610)^16^ and rhesus macaques (GEO: GSE196929, GSE228032).^17^ Preprocessing and integrated analysis of the raw data were conducted via the Seurat R package workflow (v5.3.0; https://github.com/satijalab/seurat). Stringent quality control measures were applied to all cells, retaining only those with more than 500 detected genes (nFeature_RNA > 500) and a mitochondrial gene percentage below 25%. Doublets were predicted and removed via the DoubletFinder R package (v2.0.4; https://github.com/chris‐mcginnis‐ucsf/DoubletFinder), with the expected doublet rate set to 7.5%. Following data normalization, the 3000 most highly variable genes were selected for downstream analyses. To integrate the datasets and correct for technical batch effects, the Harmony algorithm was applied. Linear dimensionality reduction was performed by principal component analysis (PCA), and the top 30 principal components (PCs) were used to generate uniform manifold approximation and projection (UMAP) for nonlinear visualization. Graph‐based clustering was conducted at a resolution of 0.5 using the FindNeighbors and FindClusters functions (Seurat v5.3.0; https://github.com/satijalab/seurat). To identify DEGs, a “pseudobulk” strategy was adopted. The expression matrices of the cells within each biological sample were aggregated, and differential analysis was performed via DESeq2 (v1.46.0; https://github.com/thelovelab/DESeq2). This process identified 1304 significantly upregulated genes post‐injury. Using the getMetaPrograms function from the GeneNMF R package (v0.9.2; https://github.com/carmonalab/GeneNMF), 10 stably expressed ‘meta‐programs’ (MPs) were subsequently identified. On the basis of functional enrichment analysis, MP3, which is closely associated with neuroinflammation and immune responses, was selected for further investigation. The microglial cell population was extracted from the integrated dataset for subtype‐specific analysis. Fifteen PCs and 2000 highly variable genes were used, with the clustering resolution set to 0.2. The marker genes for each subtype were identified via the FindAllMarkers (v5.3.0; https://github.com/satijalab/seurat) function combined with the Wilcoxon rank sum test. Gene functional enrichment analysis was performed via the Gene Ontology (GO) database. Potentially differentiated trajectories of microglia during the SCI process were inferred via Monocle3 (v1.3.7; https://github.com/cole‐trapnell‐lab/monocle3) and Slingshot (v2.7.0; https://github.com/kstreet13/slingshot). Gene expression density plots were generated via the Nebulosa R package (v1.16.0; https://github.com/powellgenomicslab/Nebulosa).

### 2.13 Bulk RNA sequencing and analysis

At 7 days post‐injury, spinal cord tissues were harvested and total RNA was extracted using QIAzol Lysis Reagent (79306; Qiagen). RNA purity and integrity were assessed with a NanoDrop 2000 spectrophotometer (Thermo Fisher Scientific) and an Agilent 5300 Bioanalyzer (Agilent Technologies). Only samples with an RNA quality number (RQN) > 4.5 were used for library construction. RNA‐seq libraries were prepared according to the Illumina® Stranded mRNA Prep Ligation protocol (Illumina) and sequenced on an Illumina NovaSeq X Plus system to generate 150 bp paired‐end reads by Shanghai Majorbio Biopharm Technology Co., Ltd.

The raw reads were quality controlled and adapter trimmed via fastp (v0.23.4; https://github.com/OpenGene/fastp). Cleaned reads were then aligned to the mouse reference genome via HISAT2 (v2.2.1; https://daehwankimlab.github.io/hisat2/). Gene expression levels were quantified as transcripts per million reads (TPM) via RSEM (v1.3.3; https://deweylab.github.io/RSEM/). To delineate differentially expressed genes (DEGs), we employed the R‐based DESeq2 package (v1.56.1; https://bioconductor.org/packages/release/bioc/html/DEGseq.html), applying rigorous cutoff criteria of an FDR < .05 and |log2FC| ≥ 1. Subsequent functional evaluation, specifically KEGG pathway enrichment, was executed using the SciPy library in Python (FDR < .05). The overarching bioinformatics pipeline was facilitated by the Majorbio Cloud Platform (https://cloud.majorbio.com/).

### 2.14 Statistical analysis

Sample size estimation was performed via G*Power 3.1 software (Franz Faul, Universität Kiel). On the basis of preliminary data, we anticipated a 45%–50% improvement in the locomotor score of the BMS in the niacin‐treated group compared with the SCI vehicle group. Therefore, a minimum of 9 mice per experimental cohort was requisite to attain 80% statistical power at an alpha level of .05. Consequently, this precise sample size was strictly adopted for all subsequent behavioural evaluations. All the statistical analyses were performed via GraphPad Prism 9.0 (GraphPad Software). All continuous data were assessed for normality using the Shapiro–Wilk test before parametric testing. The quantitative data are presented as the means ± standard deviation (SD). To evaluate differences between two independent cohorts, variance homogeneity was initially verified via the *F*‐test. Subsequently, a two‐tailed, unpaired Student's *t*‐test or Welch's *t*‐test was deployed depending on whether the variances were equal or unequal, respectively. For multigroup evaluations, we used a one‐way analysis of variance (ANOVA) coupled with Tukey's post hoc comparisons. Furthermore, a two‐way repeated measures (RM) ANOVA was implemented to analyse longitudinal or repeated‐measure datasets, with subsequent multiple comparisons evaluated via Tukey's post hoc test. A threshold of *p* < .05 was established to indicate statistical significance.

## 3 RESULTS

### 3.1 SCI increased microglial *Hcar2* gene expression in mice and nonhuman primates

**FIGURE 1:**
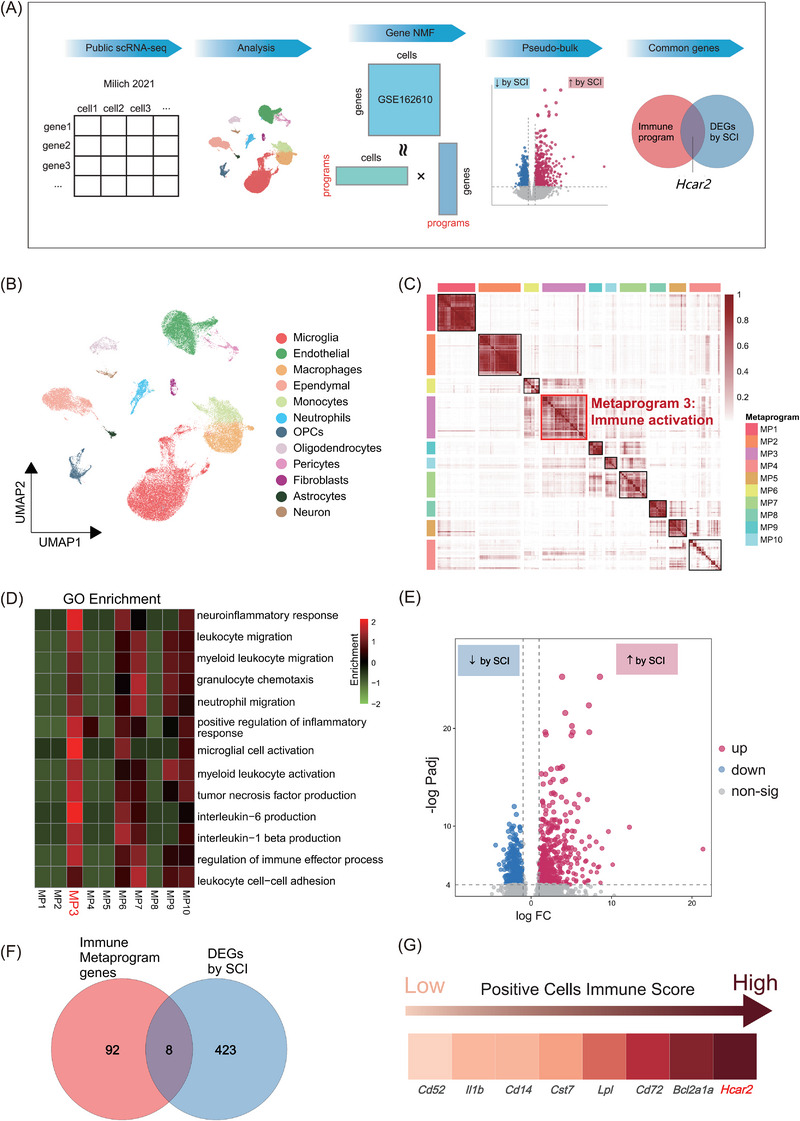
Single‐cell RNA sequencing showed cellular heterogeneity in the injured spinal cord and identified *Hcar2* as a highly expressed gene in activated microglia following SCI. (A) Schematic of the bioinformatic workflow, which integrates public scRNA‐seq data (GEO: GSE162610) and employs nonnegative matrix factorization (NMF) and pseudobulk differential gene expression (DEG) analysis. (B) UMAP visualization of 12 distinct cell types identified from 59 040 cells in the mouse spinal cord. The cell clusters are color‐coded by annotation. (C) NMF consensus matrix heatmap identifying 10 stable gene meta‐programs (MPs). MP3, the immune activation module, is highlighted. (D) Gene Ontology (GO) enrichment heatmap for genes within the top MP. MP3 is highly enriched in neuroinflammatory and immune response pathways. (E) Volcano plot of DEGs (SCI vs sham). The upregulated genes (*n* = 373; adjusted *p <* .001, log2FC > 1) are red; the downregulated genes (*n* = 297; adjusted *p <* .001, log2FC < ‐1) are blue. (F) Venn diagram illustrating the intersection of significantly upregulated DEGs and the immune meta‐programme (MP3) signature. (G) Key genes, including *Hcar2*, identified at the intersection of MP3 and upregulated DEGs, contributing to the ‘immune score’.

To identify the key genes mediating the neuroinflammatory response after SCI in mice, we conducted an integrated bioinformatic analysis of a publicly available single‐cell RNA sequencing (scRNA‐seq) dataset (GSE162610, Figure 1A). This dataset, comprising 59 040 cells, included 12 distinct cell types in the injured spinal cord (Figure 1B). To delineate the biological programs underlying this complex transcriptional landscape, we further performed two complementary analyses. First, we identified 10 distinct gene meta‐programs (MPs) via a nonnegative matrix factorization (NMF) method (Figure 1C, Table S2). Among them, MP3 was the most strongly associated with neuroinflammation and immune response pathways (Figure 1D) and was predominantly enriched in the microglial population (Figure S1A,B), the primary mediators of posttraumatic neuroinflammation.^18^, ^19^, ^20^ Second, pseudobulk differential gene expression (DEG) analysis revealed 373 upregulated genes in SCI mice compared with control mice (Figure 1E). Intersecting the SCI‐upregulated DEGs with the MP3 gene signature showed 8 shared genes, including *Cd52, II1b, Cd14, Cst7, Lpl, Cd72, Bcl2a1a* and *Hcar2*, with *Hcar2* showing the highest enrichment score (Figure 1F,G).

**FIGURE 2:**
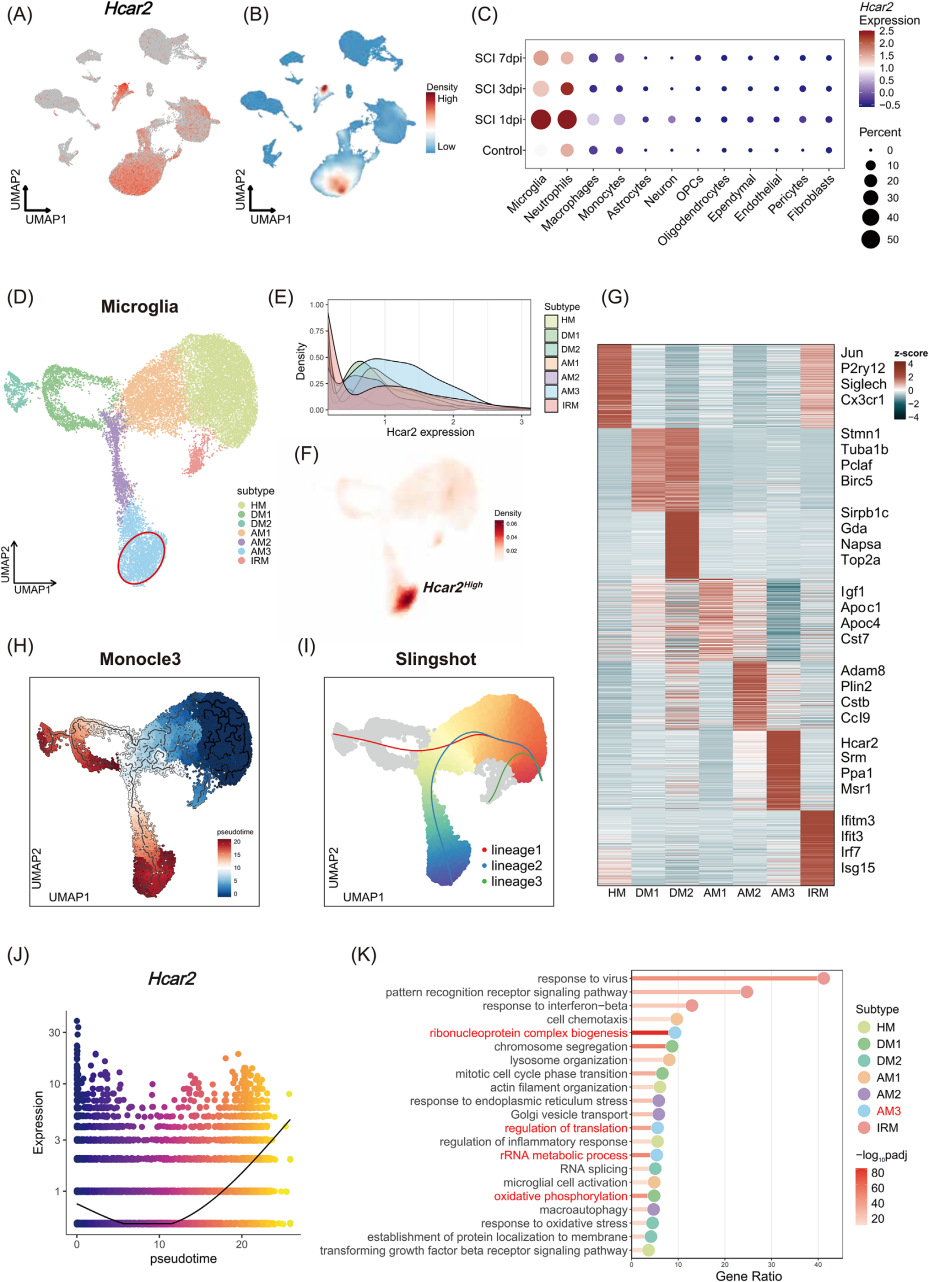
*Hcar2* was specifically upregulated in an activated microglial subtype (AM3) following SCI. (A) UMAP plot showing *Hcar2* expression, primarily localized to immune cell clusters. (B) Kernel density plot confirming high Hcar2 expression density within microglia and neutrophil populations. (C) Dotplot illustrating *Hcar2* expression levels and percentage of expression across all identified cell types at 1‐, 3‐, and 7‐day post‐injury (dpi) compared to sham controls. (D) UMAP plot of the reclustered microglial population, identifying 7 distinct subtypes: homeostatic microglia (HM), differentiating microglia (DM), activated microglia (AM1‐3), and interferon‐related microglia (IRM). (E and F) Ridge plot (E) and density plot overlay (F) showing *Hcar2* expression is highly and specifically concentrated within the AM3 microglial subtype. (G) Heatmap displaying scaled expression of selected marker genes defining each microglial subtype. (H and I) Pseudotime trajectory analysis using Monocle3 (H) and Slingshot (I), both of which identify a differentiation path from HM toward the AM3 subtype. (J) *Hcar2* expression plotted along the pseudotime trajectory, demonstrating progressive upregulation during differentiation. (K) Gene ontology (GO) enrichment analysis for marker genes of each microglial subtype.

We next sought to characterize the spatial‒temporal dynamic expression of *Hcar2* at single‐cell resolution. Given the shared myeloid origin of resident microglia and infiltrating monocyte‐derived macrophages, discriminating their respective contributions to SCI pathology is essential. In our scRNA‐seq analysis, we resolved these populations using established canonical markers (*Tmem119*/*P2ry12*/*Cx3cr1* for resident microglia; *Lyz2*/*Ms4a7*/*Gpnmb* for infiltrating monocyte‐derived macrophages) (Figure S2A).^21^, ^22^ Notably, injury‐induced upregulation of *Hcar2* was predominantly confined to the resident microglial cluster. Further analysis of the scRNA‐seq dataset revealed that *Hcar2* was expressed primarily in immune cell populations (Figure 2A), with kernel density estimation plots indicating enrichment in microglia and neutrophils (Figure 2B). *Hcar2* expression was increased in microglia at 1, 3 and 7 days post‐SCI compared with that in control microglia (Figure 2C). To further investigate the cellular heterogeneity of this response, high‐resolution subclustering of the microglial population revealed 7 distinct microglial subtypes, including homeostatic microglia (HM), differentiating microglia (DM), activated microglia (AM), and interferon‐related microglia (IRM) (Figure 2D). Notably, *Hcar2* was selectively enriched in the activated microglia 3 (AM3) subtype (Figure 2E–G). Furthermore, pseudotime trajectory analysis revealed a clear differentiation path from the HM to the AM3 subtype, along with progressively increased expression of *Hcar2* (Figure 2H–J, Figure S2B). The functional enrichment of *Hcar2*‐high AM3 cells demonstrated the upregulation of pathways associated with RNA processing, translation regulation, and ribosome biogenesis (Figure 2K), which is consistent with a metabolically active, protein‐synthetic state characteristic of microglial activation.^23^ Finally, an integrated analysis of scRNA‐seq data from rhesus macaques confirmed conserved *Hcar2* expression patterns in activated microglia following SCI (Figure S3), supporting its evolutionary conservation as a regulator of neuroinflammation.

### 3.2 SCI increased Hcar2 protein expression in microglia in vivo and in vitro

**FIGURE 3:**
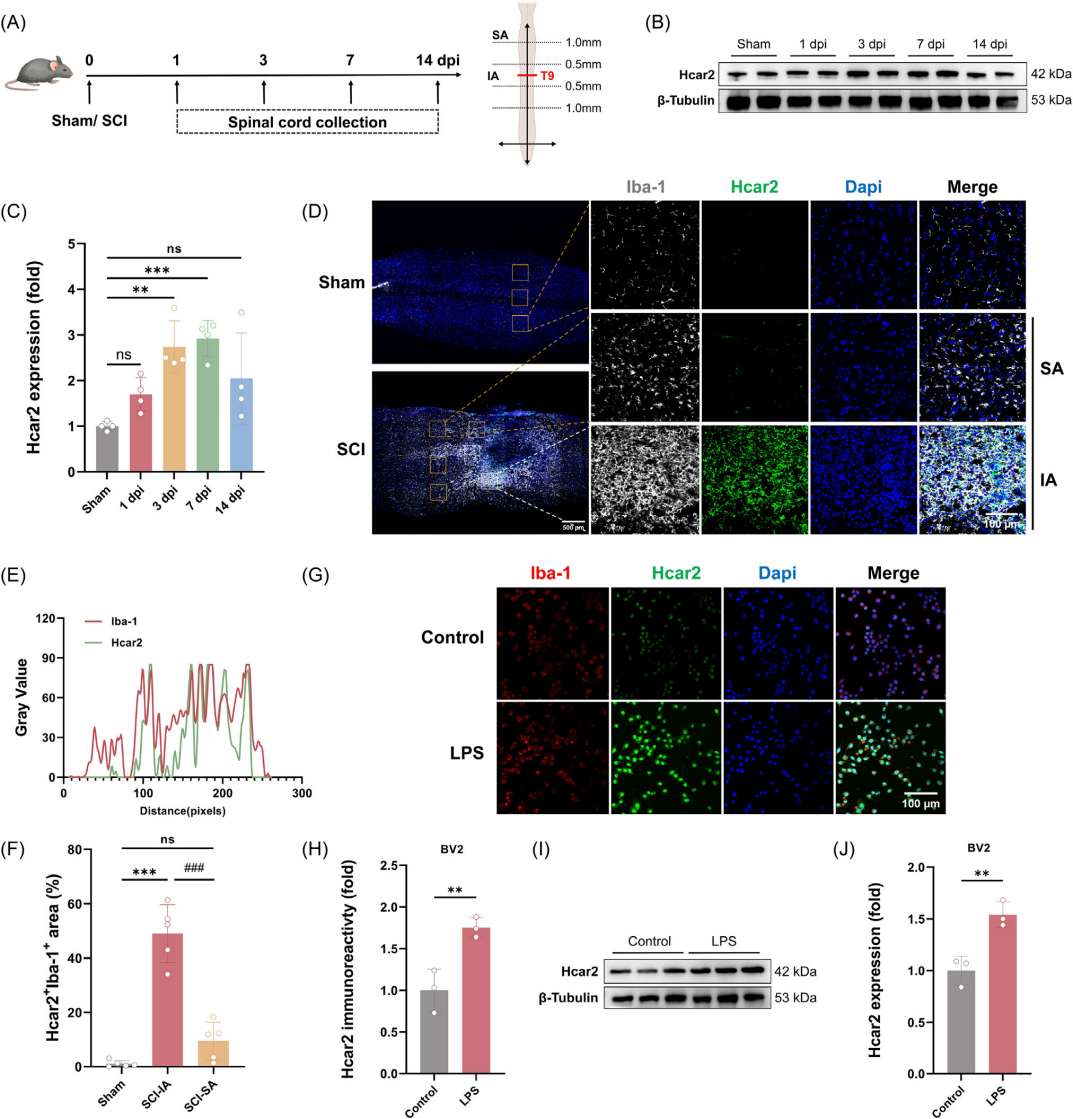
Hcar2 protein expression was upregulated in perilesional microglia following SCI. (A) Schematic timeline of the in vivo experimental design, indicating spinal cord tissue collection points (days post‐injury, dpi) and the definition of the IA and SA regions. (B) Representative Western blot analysis of Hcar2 protein levels in spinal cord lysates at 1, 3, 7 and 14 dpi. β‐Tubulin served as the loading control (*n* = 4). (C) Densitometric quantification of Hcar2 protein levels relative to those of β‐Tubulin (*n* = 4). (D) Representative confocal images of spinal cord sections at 7 dpi stained for Iba‐1 (microglia, grey) and Hcar2 (green). Nuclei were counterstained with DAPI (blue). Scale bars: 500 µm (overview), 100 µm (inset). (E) Fluorescence intensity line scan profile across a representative Iba‐1^+^Hcar2^+^ cell in the IA region, demonstrating signal colocalization (*n* = 5). (F) Quantification of the Hcar2^+^ area as a percentage of the total Iba‐1^+^ microglial area (*n* = 5). (G) Representative immunofluorescence images of BV2 cells treated with vehicle or LPS (100 ng/mL) for 24 h and stained for Iba‐1 (red) and Hcar2 (green). Scale bar: 100 µm. (H) Quantification of Hcar2 immunofluorescence intensity in BV2 cells (*n* = 3 independent experiments). (I) Representative Western blot analysis of Hcar2 protein levels in BV2 cell lysates. (J) Densitometric quantification of Hcar2 protein levels relative to those of β‐tubulin (*n* = 3 independent experiments). The data are presented as the means ± SD. Statistical significance was determined via one‐way ANOVA with Tukey's post hoc test. Normal distribution was confirmed using the Shapiro–Wilk test. **p < .01, ***p < .001 vs. Sham. ###*p <* .001 vs. SCI‐IA; ns, not significant.

To validate *Hcar2* expression in microglia following SCI, a T9 contusion SCI mouse model was established (Figure 3A). Western blot analysis of spinal cord lysates showed a time‐dependent increase in Hcar2 protein expression, beginning at 3 days post‐injury (dpi) (2.7 [.6] fold increase vs. sham; *p *= .002), peaking at 7 dpi (2.9 [.4] fold increase vs. sham; *p <* .001), and decreasing by 14 dpi (Figure 3B,C). This temporal profile was consistent with the activation kinetics of microglia and the acute inflammatory phase following SCI.^3^ Immunofluorescence analysis further confirmed an increase in Hcar2 protein expression in Iba‐1^+^ microglia in the perilesional area, a critical region implicated in reactive microgliosis and glial scar formation.^24^, ^25^ The upregulated Hcar2 signal was predominantly localized within .5 mm of the lesion core (injured area, IA), with a greater proportion of Hcar2^+^Iba‐1^+^ microglia (49.1 ± 10.6%) than within the spared area (SA; > 1 mm from the lesion centre; 9.5 ± 7.0%; *p <* .001; Figure 3D–F). To determine whether this upregulation was a direct consequence of inflammatory stimulation, BV2 microglia were treated with lipopolysaccharide (LPS), a classical TLR4 agonist.^20^ LPS exposure increased Hcar2 protein expression, as shown by both immunofluorescence (1.8 [.1]‐fold increase vs. control; *p *= .01; Figure 3G,H) and Western blot analyses (1.5 [.1]‐fold increase vs. control; *p *= .007; Figure 3I,J).

### 3.3 Deletion of *Hcar2* reversed microglial differentiation and metabolism after SCI

**FIGURE 4:**
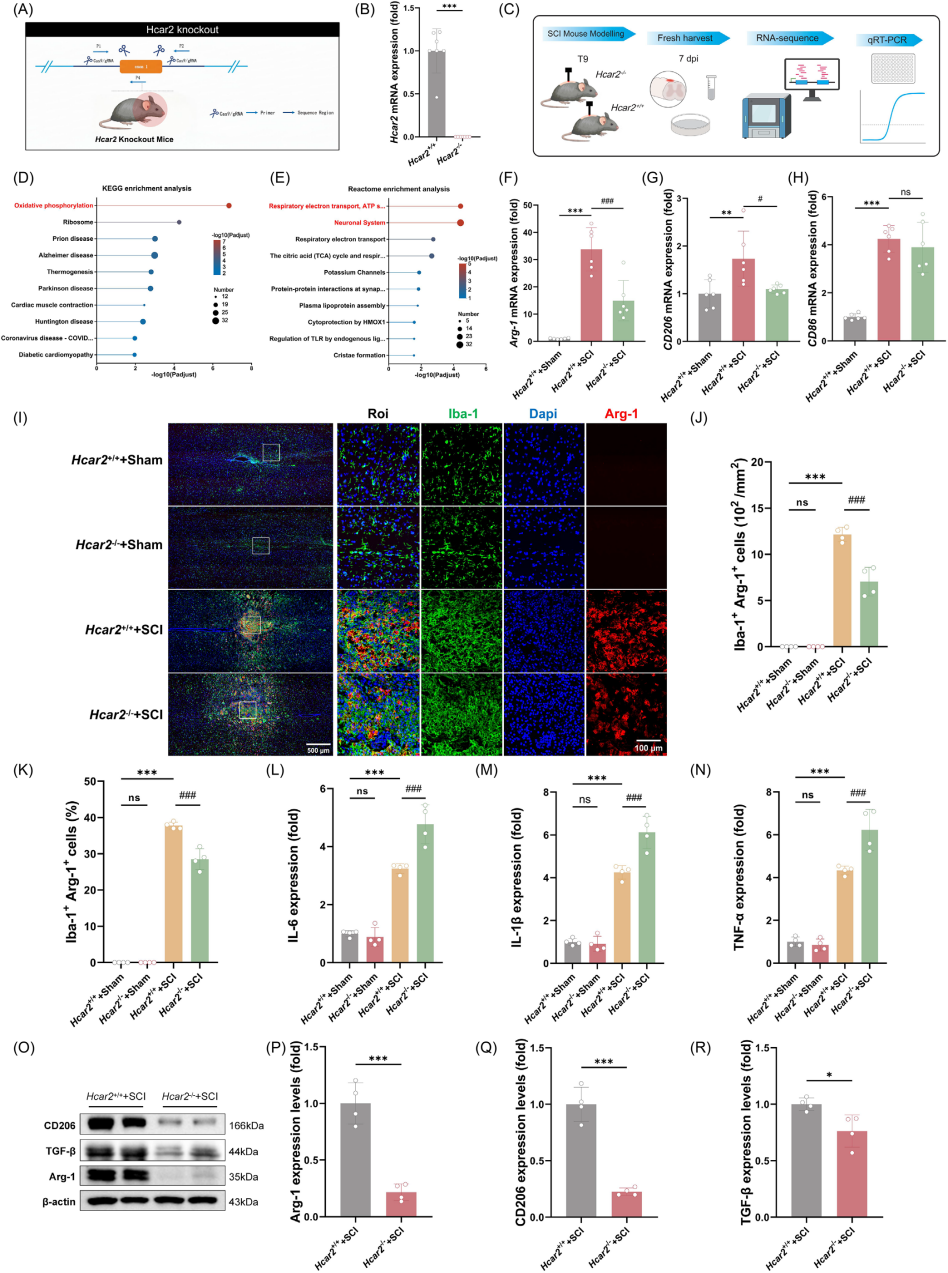
*Hcar2* deficiency induced a metabolic shift toward oxidative phosphorylation and exacerbated neuroinflammation. (A) Schematic illustration of the *Hcar2* knockout (KO) strategy. (B) qRT‒PCR confirmation of *Hcar2* mRNA expression in the spinal cords of *Hcar2* ^+/+^ and *Hcar2* ^−/−^ mice (*n* = 6). (C) Schematic workflow of the in vivo study, including SCI modelling, tissue harvesting at 7 dpi, RNA sequencing, and qRT‒PCR validation. (D and E) KEGG (D) and Reactome (E) pathway enrichment analyses of differentially expressed genes (DEGs) between *Hcar2* ^+/+^ and *Hcar2* ^−/−^ mice, highlighting the downregulation of metabolic pathways (*n* = 3). (F–H) qRT‒PCR analysis of the expression of the microglial markers *Arg1* (F), *Cd206* (G), and *Cd86* (H) in the spinal cord at 7 dpi (*n* = 6). (I) Representative images of immunofluorescence staining for Iba‐1 (green) and Arg‐1 (red) in the lesion core from *Hcar2* ^+/+^ and *Hcar2* ^−/−^ Hcar2^+/+^ mice at 7 dpi. Scale bars: 500 µm (overview), 100 µm (inset) (*n* = 4). (J, K) Quantification of Iba‐1^+^ Arg‐1^+^ cell density (J) and the percentage of Arg‐1^+^ cells within the Iba‐1^+^ population (K) (*n* = 4). (L‐N) Multiplex flow cytometric analysis of IL‐6 (L), IL‐1β (M) and TNF‐α (N) in spinal cord lysates (*n* = 4). (O) Representative Western blots of M2‐like markers (Arg‐1, CD206 and TGF‐β) (*n* = 4). (P–R) Densitometric quantification of Arg‐1 (P), CD206 (Q) and TGF‐β (R) protein levels normalized to those of β‐actin (*n* = 4). The data are presented as the means ± SD. Statistical significance was determined via one‐way ANOVA with Tukey's post hoc test. Normal distribution was confirmed using the Shapiro–Wilk test. *p < .05, **p < .01, ***p < .001 vs. *Hcar2* ^+/+^+Sham. #*p <* .05, ###*p <* .001 vs. *Hcar2* ^+/+^+SCI; ns, not significant.

Next, the *Hcar2* knockout mouse model was established to investigate the role of *Hcar2* in the injured spinal cord (Figure 4A, Figure S4A). *Hcar2* ^−/−^ mice presented a significant decrease in *Hcar2* gene expression (Figure 4B). We performed bulk RNA sequencing (RNA‐seq) on perilesional spinal cord tissue collected at 7 dpi from two SCI groups: *Hcar2* ^+/+^ + SCI and *Hcar2* ^−/−^ + SCI (Figure 4C). Loss of *Hcar2* induced 996 DEGs, including 596 upregulated genes and 400 downregulated genes (Figure S4B). KEGG analysis exhibited oxidative phosphorylation as the top pathway enriched in the comparisons (Figure 4D). Reactome enrichment analysis revealed ‘Respiratory electron transport, ATP synthesis by chemical coupling’ and ‘Neuronal System’ as the top enriched pathways (Figure 4E). In addition, gene set enrichment analysis (GSEA) showed increased enrichment of microglial differentiation in both transcriptomes (NES = 1.43, P = .03; Figure S4C), implicating *Hcar2* as a critical regulator of this process.^26^ Furthermore, we performed qRT‒PCR for representative M1/M2 polarization markers of microglia.^27^ The expression of the classical M2 (anti‐inflammatory) marker *Arg‐1* was increased in *Hcar2* ^+/+^ + SCI mice (33.8 (7.9)‐fold; *p <* .001) but was reduced in *Hcar2* ^−/−^ + SCI mice (15.0 (7.4)‐fold; *p <* .001; Figure 4F). Similarly, the M2 marker *CD206* was significantly reduced in *Hcar2* ^−/−^ mice following SCI (1.1 [.1]‐fold vs. 1.7 [.6]‐fold greater than that in the *Hcar2* ^+/+^ + SCI group; *p *= .02; Figure 4G). In contrast, although the M1 (proinflammatory) marker *CD86* was increased in *Hcar2* ^+/+^ mice following SCI, the deletion of *Hcar2* had no significant effect (Figure 4H).

We therefore proceeded to characterize the changes in anti‐inflammatory microglia by analysing the lesion sites in *Hcar2* ^−/−^ and *Hcar2* ^+/+^ mice at 7 days post‐injury. Immunofluorescence staining demonstrated an increase in the number of Arg‐1‐positive microglia in *Hcar2* ^+/+^ mice, but this increase was reduced in *Hcar2* ^−/−^ mice (28.5 [2.9] % vs. 37.8 [1.0] % of Hcar2+/+ mice; *p <* .001; Figure 4I–K). To confirm that this difference was due to impaired differentiation rather than altered cell proliferation,^28^ we quantified Iba‐1^+^/Ki‐67^+^ cells at 3 days post‐SCI and observed no significant difference between genotypes (Figure S4D–F). Multiplex flow cytometric analysis showed that the levels of the proinflammatory cytokines IL‐6 (4.8 [.7] vs. 3.2 [.2] fold of *Hcar2* ^+/+^ + Sham mice; *p <* .001; Figure 4L), IL‐1β (6.1 [.8] vs. 4.3 [.3] fold of *Hcar2* ^+/+^ + Sham mice; *p <* .001; Figure 4 M), and TNF‐α (6.2 [1.0] vs. 4.3 [.2] fold of *Hcar2* ^+/+^ + Sham mice; *p <* .001; Figure 4N) were increased in *Hcar2* ^−^/^−^ mice compared with *Hcar2*+/+ mice. Furthermore, Western blot analysis showed that the protein levels of the M2‐like markers Arg‐1 (.2 [.1] vs. 1 .2]; *p <* 0.001; Figure 4O,P) and CD206 (.2 [.03] vs. 1 [.2]; *p <* .001; Figure 4O,Q), as well as the immunoregulatory cytokine TGF‐β (.7 [.1] vs. 1 [.1]; *p = *.02; Figure 4O,R), were decreased in *Hcar2* ^−/−^ mice.

### 3.4 Niacin induced microglial metabolic reprogramming and anti‐inflammatory polarization in vitro

**FIGURE 5:**
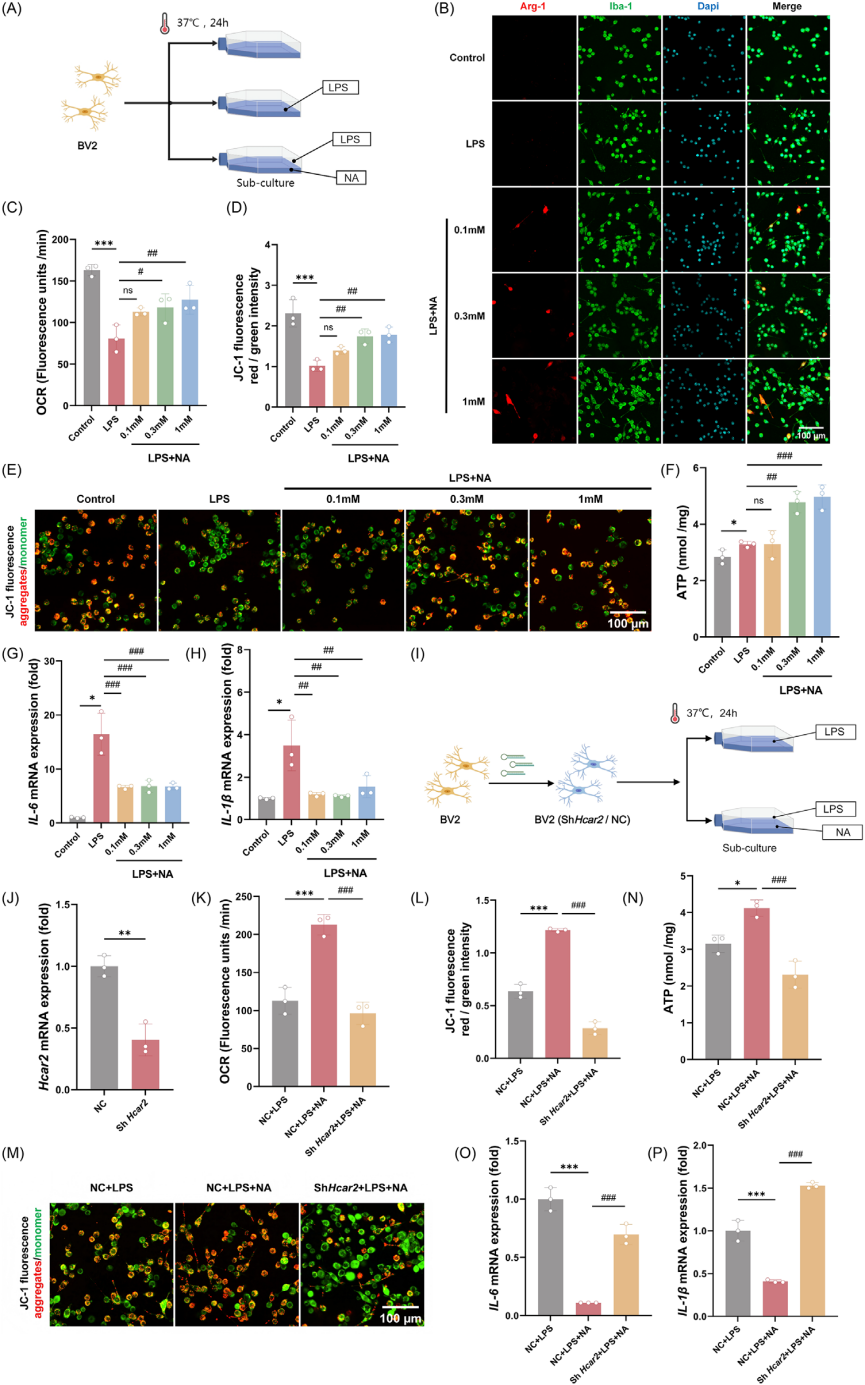
Niacin promoted an anti‐inflammatory phenotype in vitro through reprogramming microglial immunometabolism. (A) Schematic of the in vitro experimental design involving BV2 microglia challenged with LPS (100 ng/mL) and treated with niacin (NA). (B) Representative images of Iba‐1 (green) and Arg‐1 (red) immunofluorescence staining in BV2 cells treated with increasing concentrations of NA (0.1, 0.3 or 1 mM) for 24 h. Scale bar: 100 µm. (C) Quantification of OCR in BV2 cells, showing the NA‐mediated rescue of mitochondrial respiration. (D) Quantification of the red/green fluorescence intensity ratio from JC‐1 staining, which represents the relative mitochondrial membrane potential (ΔΨm). (E) Representative images of JC‐1 staining showing J‐aggregates (red, high potential) and J‐monomers (green, low potential). Scale bar: 100 µm. (F) Quantification of intracellular ATP levels. (G and H) qRT‒PCR analysis of the mRNA expression levels of the proinflammatory cytokines IL‐6 (G) and IL‐1β (H) normalized to that of β‐actin. (I) Schematic of the in vitro experimental design involving shRNA‐mediated *Hcar2* knockdown in BV2 cells followed by LPS (100 ng/mL) and NA (0.3 mM) treatment. (J) qRT‒PCR confirmation of *Hcar2* mRNA expression indicating knockdown efficiency. (K) Quantification of OCR in *Hcar2*‐knockdown BV2 cells. (L) Quantification of the red/green fluorescence intensity ratio from JC‐1 staining, representing the relative ΔΨm in *Hcar2*‐knockdown cells. (M) Representative images of JC‐1 staining in *Hcar2*‐knockdown BV2 cells showing J‐aggregates (red) and J‐monomers (green). Scale bar: 100 µm. (N) Quantification of intracellular ATP levels in *Hcar2*‐knockdown cells. (O and P) qRT‒PCR analysis of the mRNA expression levels of the proinflammatory cytokines IL‐6 (O) and IL‐1β (P) in *Hcar2*‐knockdown cells, normalized to that of β‐actin. Data are presented as the means ± SD. Statistical significance was determined via one‐way ANOVA with Tukey's post hoc test. Normal distribution was confirmed using the Shapiro–Wilk test. In A‐H, *p < .05, ***p < .001 vs. Control. #*p <* .05, ##*p <* .01, ###*p <* .001 vs. LPS; In I‐P, *p < .05, **p < .01, ***p < .001 vs. NC+LPS. ###*p <* .001 vs. NC+LPS+NA; ns, not significant; *n* = 3 independent experiments.

To further investigate the role of Hcar2 in microglial response patterns, BV2 microglia were activated with LPS and subsequently treated with NA (Figure 5A). Increasing concentrations of NA promoted anti‐inflammatory polarization, as shown by a dose‐dependent increase in the number of Arg‐1^+^ cells (Figure 5B). We next investigated whether this polarization was linked to metabolic reprogramming, a crucial feature of anti‐inflammatory (M2‐like) microglial activation.^29^ LPS treatment suppressed mitochondrial respiration, as shown by a decrease in the oxygen consumption rate (OCR; 80.9) [16.3]. vs 163.0 [6.7]; *p <* .001). However, NA treatment restored the OCR in a dose‐dependent manner (127.5 [17.3] vs. 80.9 [16.3]; *p *= .007; Figure 5C). Consistent with these findings, the mitochondrial membrane potential, as assessed via JC‐1 staining (5,5′,6,6′‐Tetrachloro‐1,1′,3,3′‐tetraethylbenzimidazolylcarbocyanine iodide staining), was decreased following LPS treatment but was reversed by NA administration (1.7 [.2] for .3 mM; *p* = .006; and 1.8 [.2] for 1 mM; *p *= .004, vs. 1.0 [.1] for LPS; Figure 5D,E;). Niacin‐treated cells (.3 and 1 mM) presented higher adenosine triphosphate (ATP) levels (4.8 [.4] nmol/mg for .3 mM; *p *= .002; 5.0 [.4] nmol/mg for 1 mM; *p <* .001; Figure 5F) than cells treated with LPS alone (3.3 [.1] nmol/mg), which is crucial for acute, rapid anti‐inflammatory responses.^30^ Furthermore, exposure to LPS upregulated the mRNA transcript levels of the pro‐inflammatory cytokines IL‐6 and IL‐1β, but NA treatment dose‐dependently reversed the induction of IL‐6 (16.5 [3.9]‐fold for LPS vs. 6.6 [.3]‐fold for .1 mM, 6.8 [1.1]‐fold for 0.3 mM, and 6.7 [.6]‐fold for 1 mM, respectively; *p <* .001 for all; Figure 5G), and IL‐1β (6.6 [.3]‐fold for LPS vs. 3.5 [1.2]‐fold for .1 mM, 1.1 [.1]‐fold for .3 mM, and 1.6 [.5]‐fold for 1 mM, respectively; *p <* .01 for all; Figure 5H).

To further validate the cell‐autonomous role of Hcar2 in microglia, we used shRNA to knock down *Hcar2* in BV2 cells (Figure 5I,J). Niacin‐induced restoration of OCR, ΔΨm and ATP production was attenuated following *Hcar2* knockdown (p < .001 for all; Figure 5K–N). Consistently, the inhibitory effect of niacin on the expression of proinflammatory cytokines IL‐6 and IL‐1β was largely abolished in Hcar2‐deficient cells (*p* < .001 for all; Figure 5O,P). These findings suggested that niacin promotes microglial immunometabolic reprogramming and anti‐inflammatory polarization predominantly through an Hcar2‐dependent mechanism.

### 3.5 Niacin improved the recovery of motor deficits in mice after SCI

We next evaluated whether pharmacological activation of Hcar2 with niacin could resolve neuroinflammation in vivo (Figure S5A). Compared with control SCI mice, NA‐treated SCI mice presented an increase in the number of Iba‐1^+^/Arg‐1^+^ cells (13.7 [2.8] vs 9.1 [1.4]; *p *= .02; Figure S5B,C) and the percentage of Arg‐1^+^ microglia (47.8 [5.6] % vs 35.0 [5.0] %; *p *= .01; Figure S5D). In parallel, the expression of the pro‐inflammatory cytokines IL‐1β (2.4 [1.0] vs 5.3 [1.3]; *p *= .01; Figure S5E) and TNF‐α (0.8 [.3] vs. 2.4 [.8], *p *= .008; Figure S5F) was decreased, whereas the IL‐6 levels did not significantly change (Figure S5G). Furthermore, western blot analysis showed upregulation of the anti‐inflammatory markers Arg‐1 (2.0 [.6] vs. 1 [.2]; *p *= .02; Figure S5H,I), CD206 (3.3 [.6] vs. 1 [.3]; *p <* .001; Figure S5H,J), and TGF‐β (1.8 [.2] vs. 1 [.2]; *p *= .003; Figure S5H,K) in NA‐treated SCI mice compared with controls.

**FIGURE 6:**
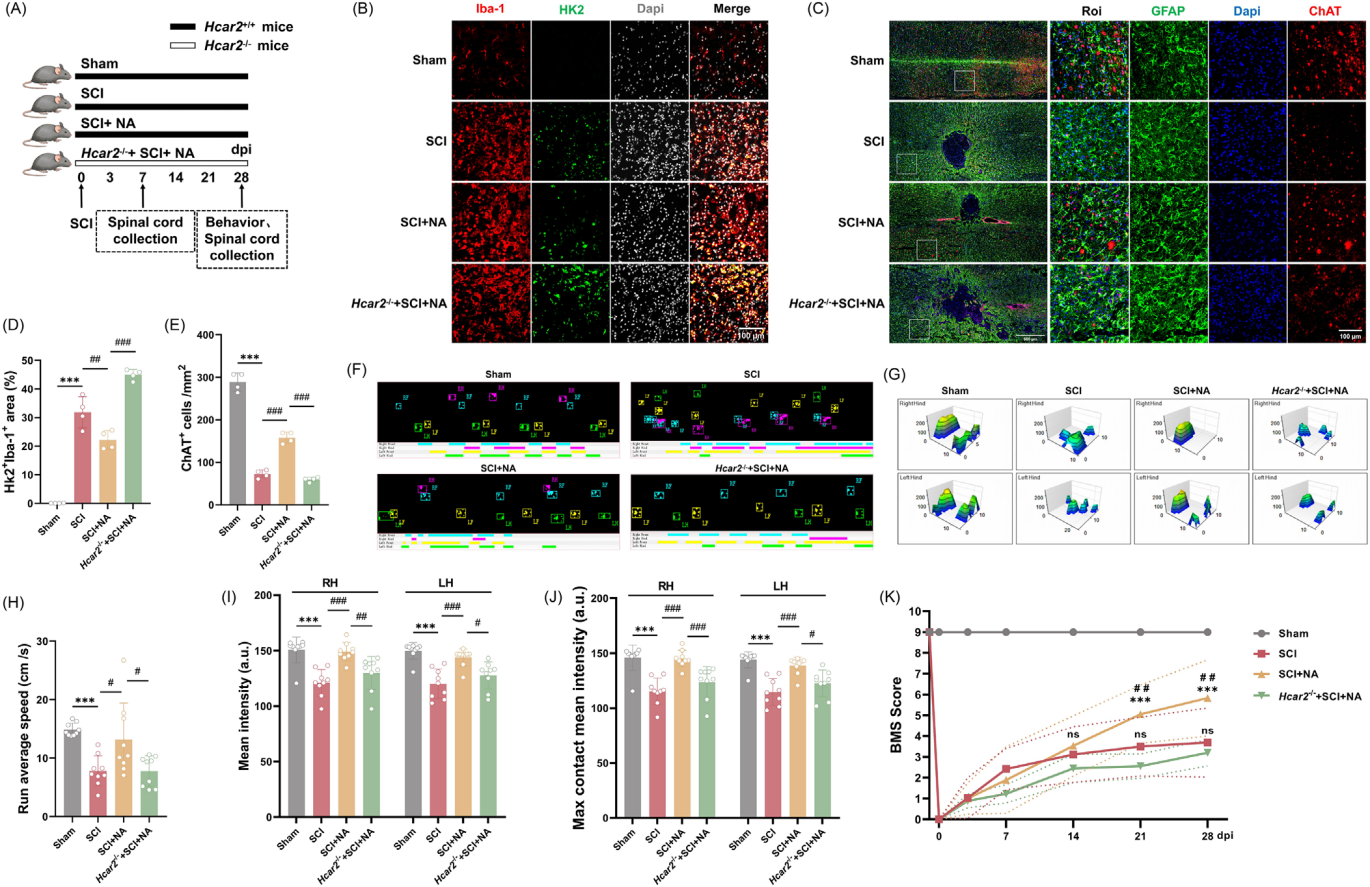
Niacin promoted neuroprotection and locomotor recovery after SCI in a Hcar2‐dependent manner. (A) Experimental timeline for niacin (NA) administration and behavioural assessments. (B) Representative double immunofluorescence images of the perilesional spinal cord at 7 dpi showing Iba‐1 (microglia, red) and HK2 (glycolytic enzyme, green) expression. Scale bar: 100 µm. (C) Representative immunofluorescence images of the perilesional spinal cord at 28 dpi showing GFAP (astrocytes, green) and ChAT (motor neurons, red) expression. The high‐magnification inset displayed a representative region rostral to the lesion core, as indicated by the white box. Scale bars: 500 µm (overview), 100 µm (inset). (D) Quantification of the HK2^+^ area within the Iba‐1^+^ microglial population at 7 dpi (*n* = 4). (E) Quantification of surviving ChAT^+^ motor neurons in the ventral horn (n = 4). (F, G) CatWalk gait analysis at 28 dpi, displaying representative footprint patterns (F) and 3D intensity maps (G). (H–J) Quantification of key gait parameters: average run speed (H), mean intensity (I), and maximum contact mean intensity (J) of the hindlimbs (*n* = 9). (K) Time course of locomotor recovery assessed by Basso Mouse Scale (BMS) scores over 28 days (*n* = 9). The data are presented as the means ± SD. Statistical significance was determined via one‐way ANOVA (D‐E, H–J) or two‐way repeated‐measures ANOVA (K) followed by Tukey's post hoc test. Normal distribution was confirmed using the Shapiro–Wilk test. ***p < .001 vs. Sham. #*p <* .05, ##*p <* .01, ###*p <* .001 vs. SCI+NA; ns, not significant.

We next investigated whether niacin treatment can promote motor recovery following SCI (Figure 6A). In vivo metabolic validation showed that niacin treatment reduced the expression of the glycolytic enzyme HK2 at 7 dpi perilesional microglia (22.2 [3.30] % vs. 31.86 [5.43] % of controls; *p* = .004). However, this metabolic rescue was abolished in *Hcar2* ^−^/^−^ mice (44.97 [1.84] % vs. 22.2 [3.30] % of SCI mice treated with NA; *p* < .001; Figure 6B,D), confirming that Hcar2‐dependent immunometabolic reprogramming is a potential mechanism following SCI. Paralysis results from the loss of spinal cord ventral horn motor neurons, particularly cholinergic (ChAT^+^) neurons, after SCI.^31^ At 28 days post‐injury, SCI mice presented a decrease in the number of ChAT^+^ cells, whereas niacin treatment preserved their number (158.0 [13.1] vs. 72.5 [9.4] for SCI; *p <* .001). This neuroprotective effect was abolished in Hcar2 knockout mice (Figure 6C,E). Motor function was further measured via Catwalk 3D gait analysis and the Basso Mouse Scale (BMS) at 28 days post‐injury.^31^ Catwalk analysis showed that niacin treatment restored key locomotor parameters, including average run speed (13.2 [6.3] cm/s vs. 7.8 [2.6] cm/s for SCI; *p *= .01; Figure 6F–H), mean paw contact intensity (*p <* .001 for right hindlimbs and left hindlimbs; Figure 6I), and maximum contact mean intensity (*p <* .001 for right hindlimbs and left hindlimbs; Figure 6J). BMS corroborated this improvement, as niacin‐treated mice presented improved locomotor performance beginning at 21 days post‐injury (BMS: 5.1 ± 1.4 vs. 3.5 ± 1.4 for SCI; *p *= .01), with a mean BMS score of 5.8 ± 1.8 on day 28 post‐injury (5.8 [1.8] vs. 3.7 [1.7] for SCI; *p *= .004; Figure 6K). However, the beneficial effects of niacin on motor performance were abolished in *Hcar2* ^−^/^−^ mice, suggesting that the neuroprotective and functional benefits of niacin require Hcar2 signalling (Figure 6F–K).

## 4 DISCUSSION

In the present study, we identified the niacin receptor Hcar2 as a pivotal regulator of the neuroinflammatory response following spinal cord injury (SCI). Our bioinformatic and in vivo data demonstrated that Hcar2 is acutely upregulated in microglia post‐SCI, where it governs metabolic reprogramming towards oxidative phosphorylation. This metabolic shift was essential for promoting the polarization of microglia from a pro‐inflammatory state to an anti‐inflammatory, reparative (M2‐like) phenotype. Consequently, pharmacological activation of Hcar2 with niacin attenuated neuroinflammation, enhanced neuroprotection, and promoted motor function recovery in an Hcar2‐dependent manner.

The devastating, permanent functional deficits following SCI are not solely caused by primary mechanical trauma but are, to a large extent, driven by a prolonged secondary injury cascade.^32^ A dysregulated and persistent neuroinflammatory response, dominated by activated microglia, is a hallmark of this secondary pathology and constitutes a primary barrier to endogenous repair.^33^ Therefore, the activation state of these cells is a critical determinant of the pathological outcome. Our study identified Hcar2 as a key regulator of this process. We demonstrated that genetic deletion of Hcar2 impairs adaptive polarization, resulting in significantly blunted expression of anti‐inflammatory markers and an exacerbated proinflammatory environment, confirming the critical role of Hcar2 in the acute inflammatory response following SCI.

A core finding of this study was the critical link between Hcar2 signalling, metabolic reprogramming, and microglial functional polarization. The functional polarization of microglia is inextricably linked to their metabolic state.^26^ Pro‐inflammatory (M1‐like) microglia typically rely on aerobic glycolysis, whereas anti‐inflammatory (M2‐like) reparative functions are sustained by intact mitochondrial respiration, including oxidative phosphorylation (OXPHOS) and the tricarboxylic acid cycle.^34^, ^35^ Our study provides clear evidence that Hcar2 regulates this metabolic switch. In vivo, RNA‐seq revealed that Hcar2 deletion significantly altered pathways related to oxidative phosphorylation and respiratory electron transport, ATP synthesis by chemiosmotic coupling. Furthermore, our in vitro experiments demonstrated that niacin‐mediated Hcar2 activation directly reversed LPS‐induced mitochondrial dysfunction, restoring the OCR, ΔΨm and cellular ATP levels. Hcar2 is a G protein‐coupled receptor known to inhibit adenylate cyclase and reduce the level of intracellular cyclic adenosine monophosphate (cAMP). The reduction in cAMP can, in turn, modulate key metabolic sensors such as protein kinase A (PKA) and adenosine monophosphate‐activated protein kinase (AMPK), both of which are central regulators of mitochondrial biogenesis and OXPHOS function.^10^, ^36^, ^37^, ^38^ Although the precise downstream of Hcar2 cascades remain to be elucidated, Hcar2 may orchestrate microglial metabolic reprogramming via classical Gi/o‐cAMP‐PKA/AMPK axes or emerging β‐arrestin‐dependent signalling.^38^, ^39^ Future targeted mechanistic studies are warranted to definitively establish their causal roles in this process following SCI.

Given its critical role as a metabolic‐immune node, Hcar2 represents a highly attractive therapeutic target. Hcar2 can be activated by endogenous metabolites, notably the ketone body β‐hydroxybutyrate (BHB), linking systemic metabolism to CNS immunity.^9^, ^40^ Crucially, Hcar2 is also a high‐affinity receptor for niacin (vitamin B3).^11^ Niacin is an FDA‐approved drug with a long history of clinical safety and, importantly, effectively crosses the blood‒brain barrier.^41^ This established pharmacological profile provides a strong theoretical basis for ‘drug repurposing’—the use of niacin to harness the Hcar2 pathway therapeutically after SCI.^42^ Our study revealed that the systemic administration of niacin after SCI provides significant neuroprotection and promotes motor function recovery beginning at 21 days post‐injury. The translation of early neuroprotective benefits into macroscopic functional recovery requires a temporal lag, as cellular improvements initiated in the early stages following SCI,^43^ but require additional time to manifest as functional gains. These protracted processes rely heavily on adaptive neural circuit reorganization and synaptic plasticity.^2^, ^44^ Niacin treatment not only promoted a beneficial M2‐like microglial phenotype but also improved the survival of cholinergic motor neurons, resulting in substantial, durable improvements in motor function. Robert M. Grumbles et al. demonstrated that the loss of cholinergic motor neurons is a primary driver of muscle denervation and dysfunction in human SCI.^45^ Furthermore, pharmacological interventions that promote the survival of these neurons have been shown to directly improve BMS scores in rodent models.^46^, ^47^ Although our findings indicated niacin promotes neuronal survival indirectly via microglia Hcar2 following SCI, whether niacin also exerts direct neuroprotective effects on neurons remains to be determined.

Mitochondrial dysfunction and excessive reactive oxygen species are key triggers for inflammasome activation and subsequent pyroptosis, characterized by IL‐1β release and cellular swelling.^48^ Our data demonstrated that niacin suppresses IL‐1β expression and attenuates macroscopic cellular swelling and tissue necrosis (Figure S4G). Although niacin is widely recognized as a precursor for nicotinamide adenine dinucleotide (NAD^+^) biosynthesis essential for cellular energy homeostasis and survival, the complete loss of its therapeutic efficacy in *Hcar2* ^−^/^−^ SCI mice indicates that its beneficial effects in this acute injury context are predominantly mediated by receptor activation rather than a generalized NAD^+^ increase. This receptor‐specific mechanism is further supported by recent studies showing that the anti‐inflammatory and neuroprotective properties of niacin in the central nervous system critically depend on Hcar2 engagement.^15^, ^41^ This finding aligns with growing evidence supporting Hcar2 activation as a neuroprotective strategy in other CNS pathologies^14^, ^15^, ^40^, ^49^, ^50^ and validated Hcar2 as a specific and highly translatable target for SCI treatment.

The present study has several limitations. First, although in‐vitro microglia‐specific *Hcar2* knockdown and in‐vivo global *Hcar2* knockout demonstrated the neuroprotective effects of niacin following SCI, microglia‐specific conditional knockout models in vivo are required to definitively confirm that these effects are mediated through microglial Hcar2 rather than other infiltrating immune populations. Second, our findings demonstrated that niacin is effective during both the acute (7 dpi) and subacute (28 dpi) phases; however, its long‐term impact on chronic pathology remains to be fully elucidated. Third, our histological assessments primarily focused on ventral horn ChAT^+^ motor neurons to align with the observed motor recovery, whether niacin confers neuroprotection to dorsal horn sensory neurons, thereby alleviating sensory deficits, requires further investigation. Finally, while supported by non‐human primate data, the lack of direct validation in human SCI biospecimens is still required to establish potential translational relevance. To more rigorously establish its true translational potential, future studies should evaluate the efficacy of delayed niacin administration (e.g., 24–72 h post‐SCI).

## 5 CONCLUSION

Our study identified Hcar2 as a pivotal regulator that promotes a reparative, anti‐inflammatory phenotype of microglia following SCI, likely through the reprogramming of immune metabolism. Furthermore, we validated niacin, an FDA‐approved Hcar2 agonist, as a translatable therapeutic strategy that enhances neuroprotection and improves motor function recovery. Collectively, these findings not only reveal a novel mechanism involved in the pathophysiology of SCI but also provide preclinical evidence supporting Hcar2 as a promising target for clinical intervention after SCI.

## AUTHOR CONTRIBUTIONS

**Hua Du**: Conceptualization; methodology; validation; formal analysis, investigation; data curation; writing—original draft; project administration. **Lingnian Zeng**: Methodology; software; validation; formal analysis; investigation and data curation. **Chan Liu**: Methodology; software; validation; formal analysis and investigation. **Huyao Zhou**: Resources. **Xia Wang**: Resources. **Qing Ai**: Resources. **Jinpiao Zhu and Nong Xiao**: Writing—review & editing; supervision; project administration and funding acquisition.

## ETHICS STATEMENT

This study was carried out in accordance with the principles of the Basel Declaration and recommendations of the US NIH with Specific Pathogen Free conditions. All animal procedures were conducted in strict adherence to the ethical guidelines established by the Animal Care and Use Committee of Chongqing Medical University (CQMU) and were approved by the CQMU Animal Care and Use Committee (No. IACUC‐CQMU‐2023‐0423). Efforts were made to minimize the number of animals used and to alleviate any potential suffering.

## CONFLICT OF INTEREST STATEMENT

The authors declare no conflicts of interest.

## Supporting information



Supporting Information

## Data Availability

The datasets generated and analysed during the current study are available from the corresponding author upon reasonable request. The RNA‐seq data have been deposited in the NCBI Sequence Read Archive (SRA) under accession number PRJNA1441145. Publicly available single‐cell RNA sequencing data used in this study were obtained from the Gene Expression Omnibus (GEO) under accession number GSE162610, GSE196929 and GSE228032.
