# Supplementary material for: Niacin promotes motor function recovery after spinal cord injury via Hcar2‐dependent microglia immunometabolic regulation
Source: Clin Transl Med. 2026 May 1;16(5):e70683. doi: 10.1002/ctm2.70683 (PMC13135113; doi:10.1002/ctm2.70683)
Supplement: Supplementary file 1 — Supporting Information [file CTM2-16-e70683-s001.docx]

**Hua Du et al., Niacin promotes motor function recovery after spinal cord injury via Hcar2-dependent microglia immunometabolic regulation**

**Supplemental Tables and Figures**

**Table S1 The sequence of the primer**

| **Gene** | **Forward primer (5ʹ–3ʹ)** | **Reverse primer (5ʹ–3ʹ)** |
| --- | --- | --- |
| *Hcar2* | TGAGGCAGAGACAGATGGACAGAC | GAGAAGCCAGAAGATGCGGATGC |
| *Arg-1* | GTGAAGAACCCACGGTCTGT | GCCAGAGATGCTTCCAACTG |
| *CD206* | CTTCGGGCCTTTGGAATAAT | TAGAAGAGCCCTTGGGTTGA |
| *CD86* | ATATGACCGTTGTGTGTGTTCTGGA | AGGGCCACAGTAACTGAAGCTGTAA |
| *IL-6* | CCAGAAACCGCTATGAAGTTCC | GTTGGGAGTGGTATCCTCTGTGA |
| *IL-1β* | GTTCCCATTAGACAACTGCACTACAG | GTCGTTGCTTGGTTCTCCTTGTA |
| *TNF-α* | CCCCAAAGGGATGAGAAGTTC | CCTCCACTTGGTGGTTTGCT |
| *β-actin* | CGCACCACTGGCATTGTCAT | TTCTCCTTGATGTCACGCAC |

**Table S2 Representative biological descriptions of NMF meta-programs**

| **Meta-programs** | **Biological functions and summary descriptions** |
| --- | --- |
| MP1 | Cilium motility/assembly |
| MP2 | Endothelial development / Angiogenesis |
| MP3 | Neuroinflammation / Immune activation |
| MP4 | Cytoskeleton regulation / Migration |
| MP5 | Glial differentiation / Synaptic regulation |
| MP6 | Inflammatory / Cytokine response |
| MP7 | Leukocyte chemotaxis / Adhesion |
| MP8 | Oligodendrocyte differentiation / Myelination |
| MP9 | Neutrophil activation / Chemotaxis |
| MP10 | Mononuclear migration / Inflammatory regulation |


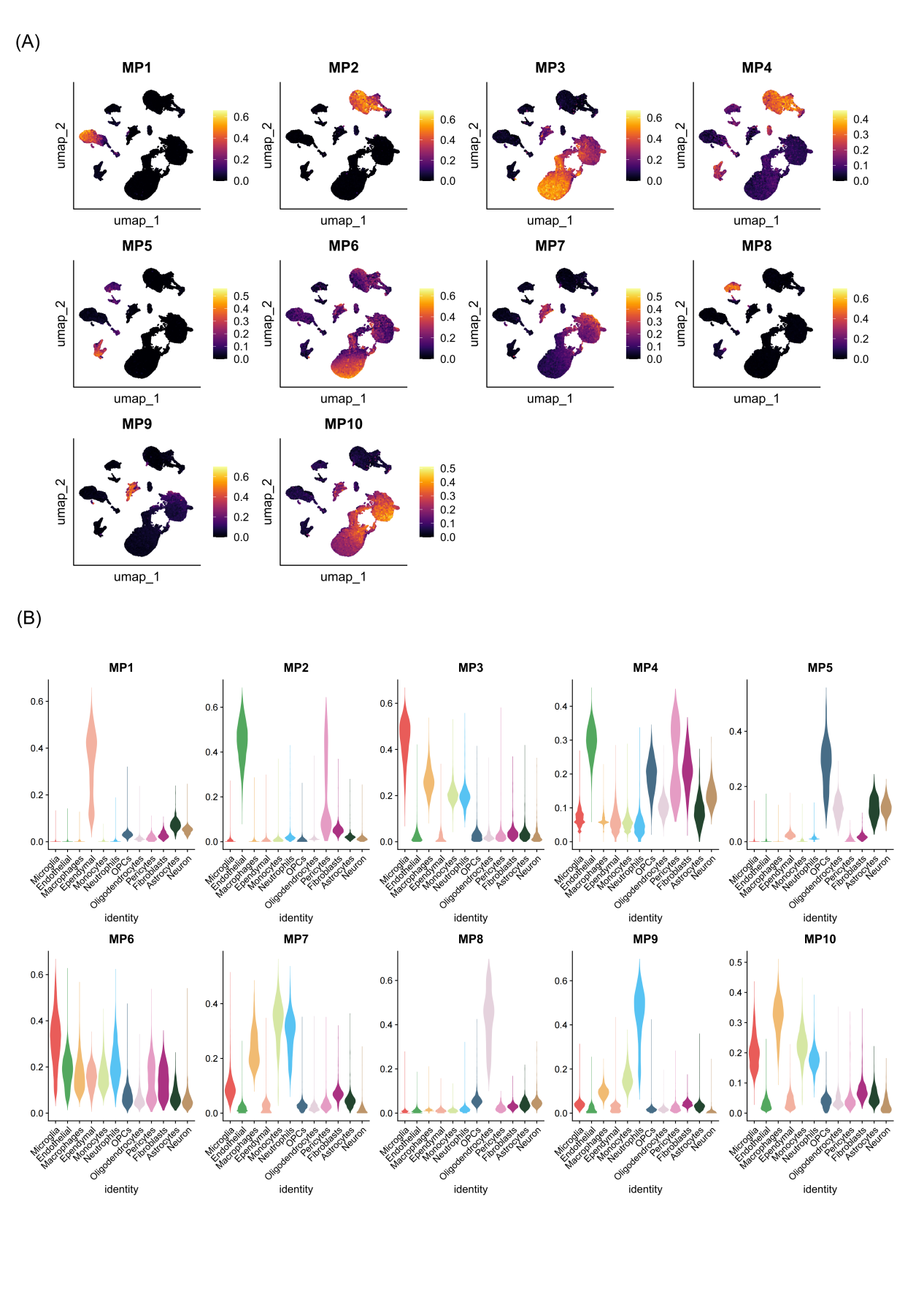


**Fig. S1. Distribution of gene meta-programs (MPs) following SCI. (A) UMAP feature plots showing the activity scores and spatial distribution for all 10 identified meta-programs (MP1–MP10) across all cell clusters. (B) Violin plots illustrating the expression distribution and cell-type specificity of each meta-program (MP1–MP10) across the 12 annotated cell types.**


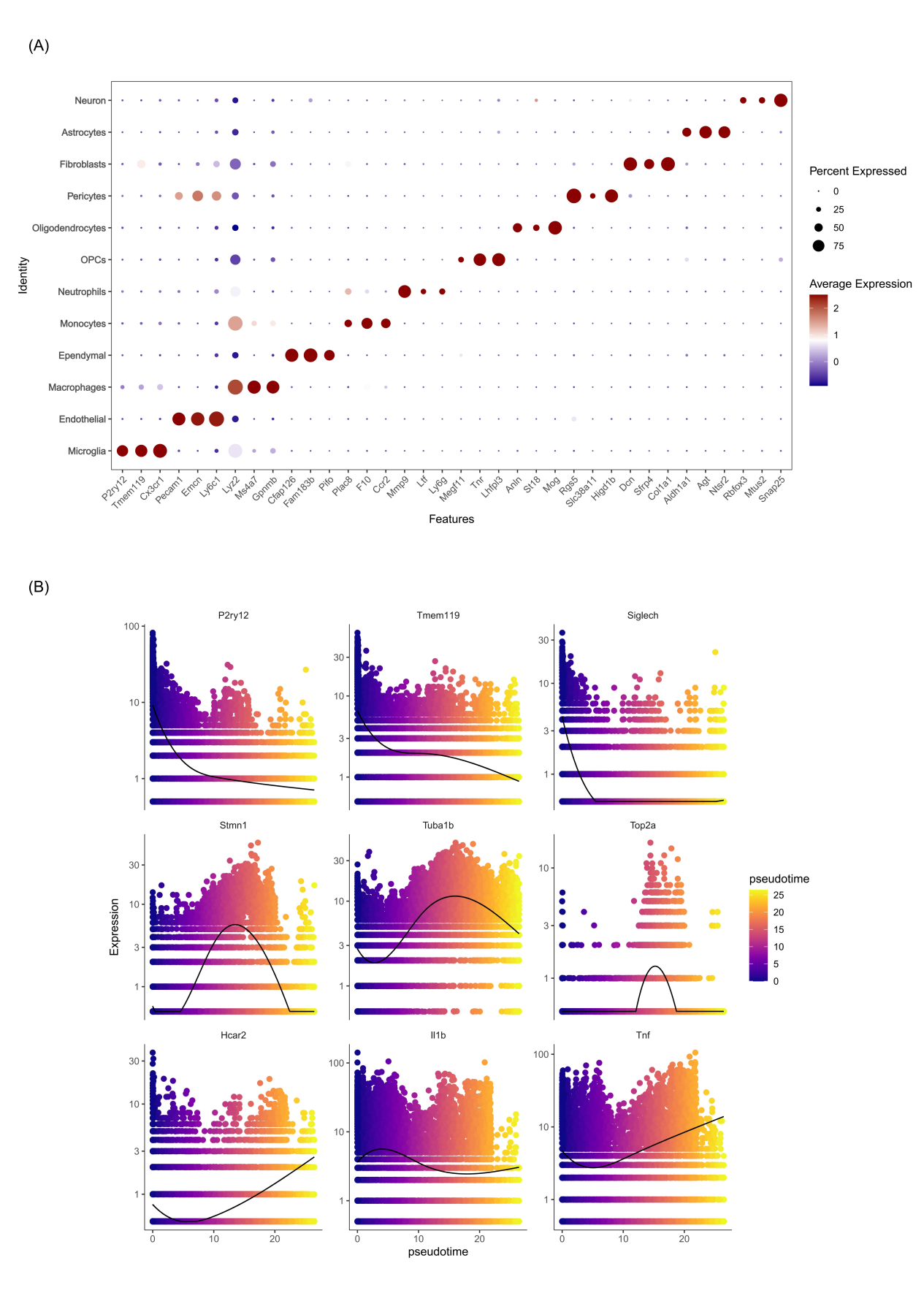


**Fig. S2. Pseudotime analysis showed dynamic expression of Hcar2 and other key genes during microglial activation.** (A) Dot plot illustrating the expression of canonical marker genes used to distinguish resident microglia (Tmem119, P2ry12, Cx3cr1) from infiltrating monocyte-derived macrophages (Lyz2, Ms4a7, Gpnmb) and other distinct cellular populations in the scRNA-seq dataset. (B) Scatter plots showing the expression of selected genes (y-axis) plotted against the microglial pseudotime trajectory (x-axis) derived in Figure 2. Each dot represented a single cell and is colored by its pseudotime value (0 = homeostatic state). The black line indicated the smoothed expression trend.


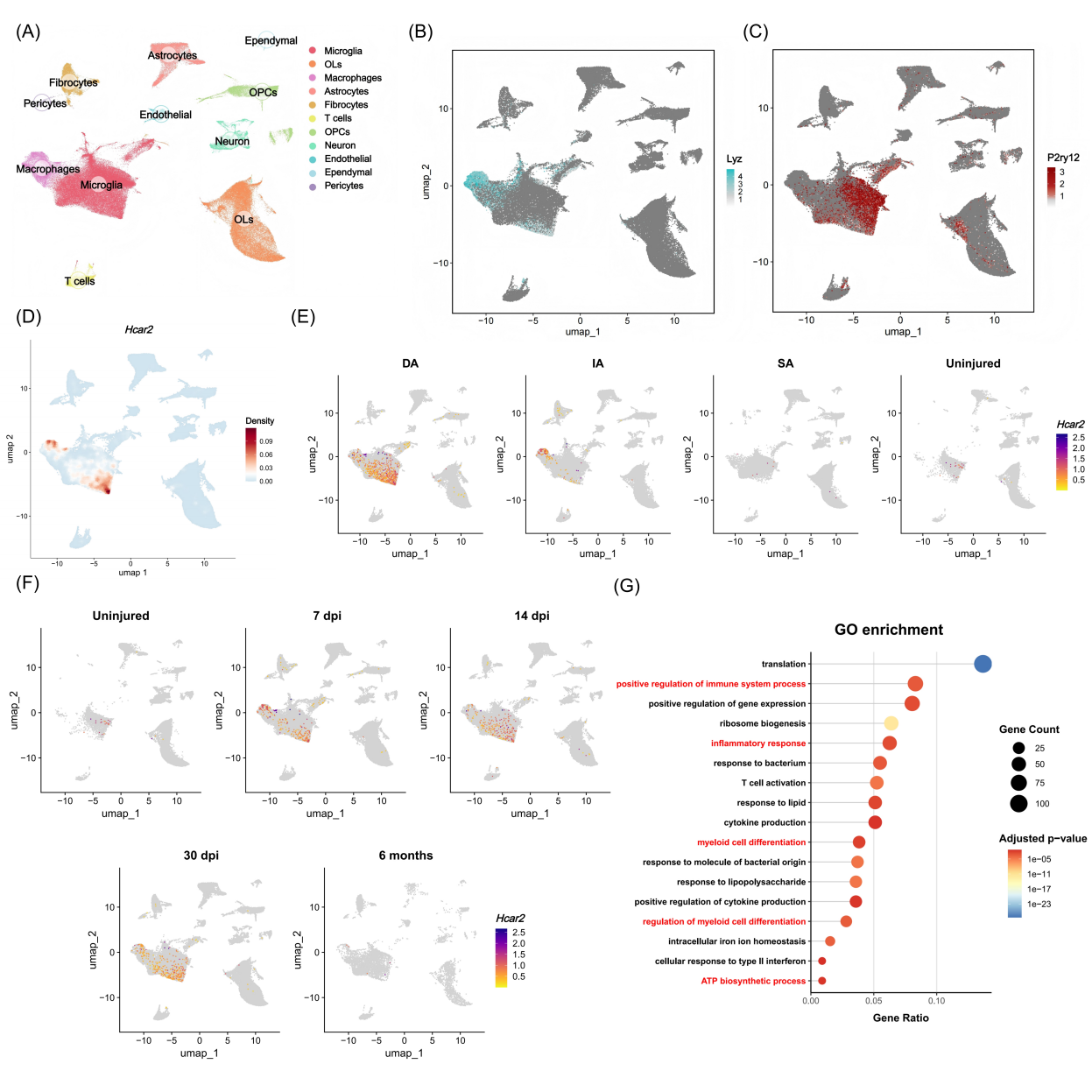


**Fig. S3. Cross-species validation of *Hcar2* upregulation in rhesus macaques following SCI.** (A) UMAP visualization of 11 distinct cell types identified from integrated scRNA-seq data (197,181 cells) of rhesus macaque spinal cords. (B-D) UMAP feature plots showing the expression distribution of the macrophage marker LYZ (B), the microglial marker *P2RY12* (C), and *HCAR2* (D), demonstrating predominant *HCAR2* expression in microglia. (E) UMAP feature plots showing spatial *HCAR2* expression across pathologically defined regions: Degenerative Area (DA), Injured Area (IA), Spared but Activated area (SA), and Uninjured. (F) UMAP feature plots illustrating the temporal upregulation of *HCAR2* expression in microglia at 7-, 14-, 30- days post-injury (dpi), and 6 months, compared to uninjured controls. (G) Gene Ontology (GO) enrichment analysis of *HCAR2*-positive microglia. Dot size represents gene count, and color represents the adjusted P-value.

**
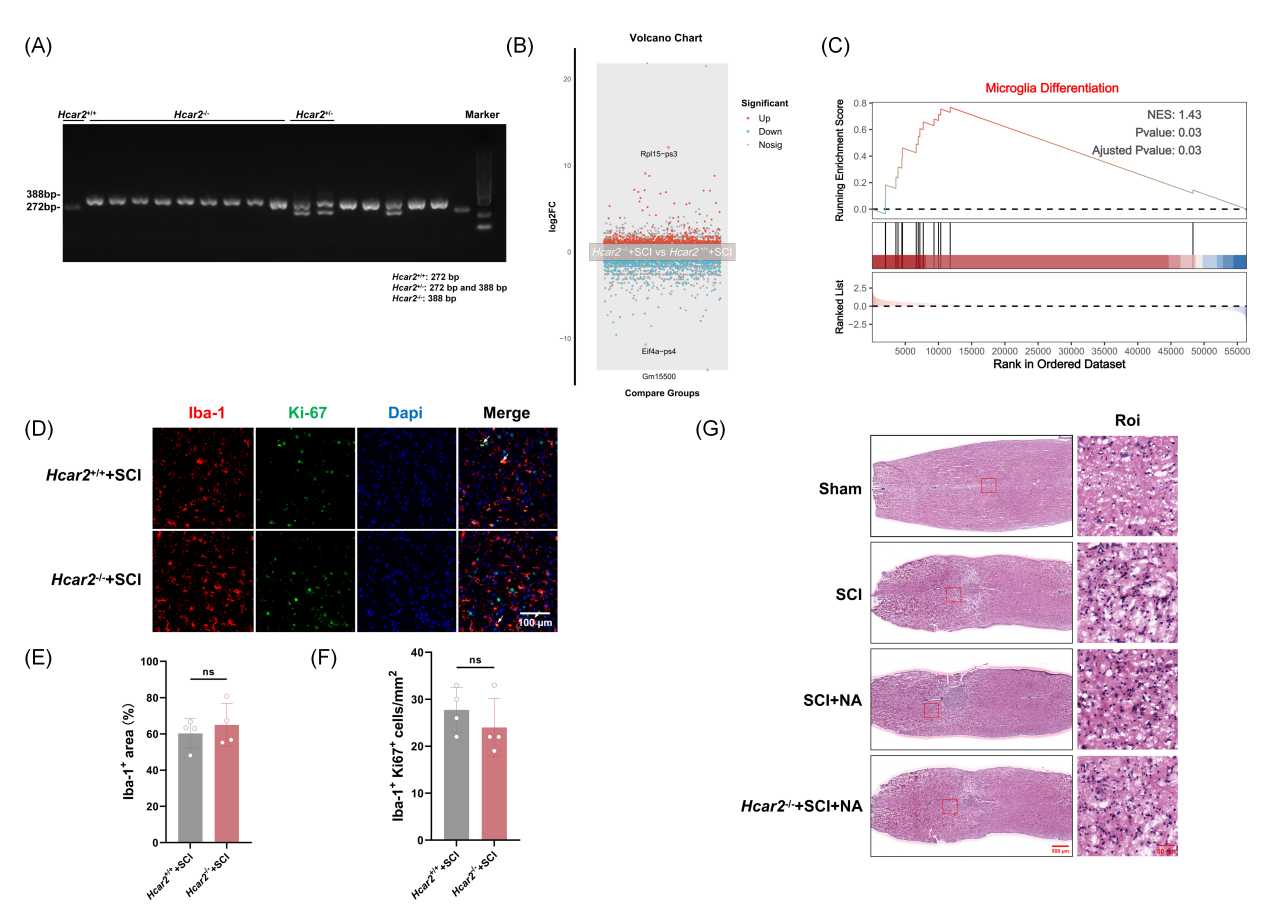

Fig. S4. Deletion of Hcar2 reversed microglial differentiation after SCI.** (A) Representative genotyping PCR gel image identifying wild-type (*Hcar2*^+/+^), heterozygous (*Hcar2*^+/-^), and knockout (*Hcar2*^-/-^) mice. (B) Volcano plot of DEGs in the perilesional spinal cord of *Hcar2*^−/−^ versus *Hcar2*^+/+^ mice at 7 dpi (n = 3). Red dots: upregulated genes (n = 596); Blue dots: downregulated genes (n = 400). (C) GSEA showing significant depletion of the ‘Microglial Differentiation’ signature in *Hcar2*^−/−^ mice (NES = 1.43, P = 0.03). (D) Representative confocal images of immunofluorescence staining in the spinal cord lesion area at 3 days post-injury (dpi) from *Hcar2*^+/+^+SCI and *Hcar2*^−/−^+SCI mice. Sections were stained for Iba-1 (microglia, red), Ki-67 (cell proliferation marker, green), and DAPI (nuclei, blue). Scale bar: 100 µm (n = 4). (E) Quantification of the Iba-1⁺ positive area (%) in the lesion area. (F) Quantification of the number of Iba-1⁺/Ki-67⁺ proliferating cells per mm² in the lesion area. (G) Representative images of Hematoxylin and Eosin (H&E) staining of longitudinal spinal cord sections at 7 dpi. Scale bars: 500 µm (overview), 50 µm (ROI). The data are presented as the means ± SD. Statistical significance was determined via a Student's t-test. ns, not significant.


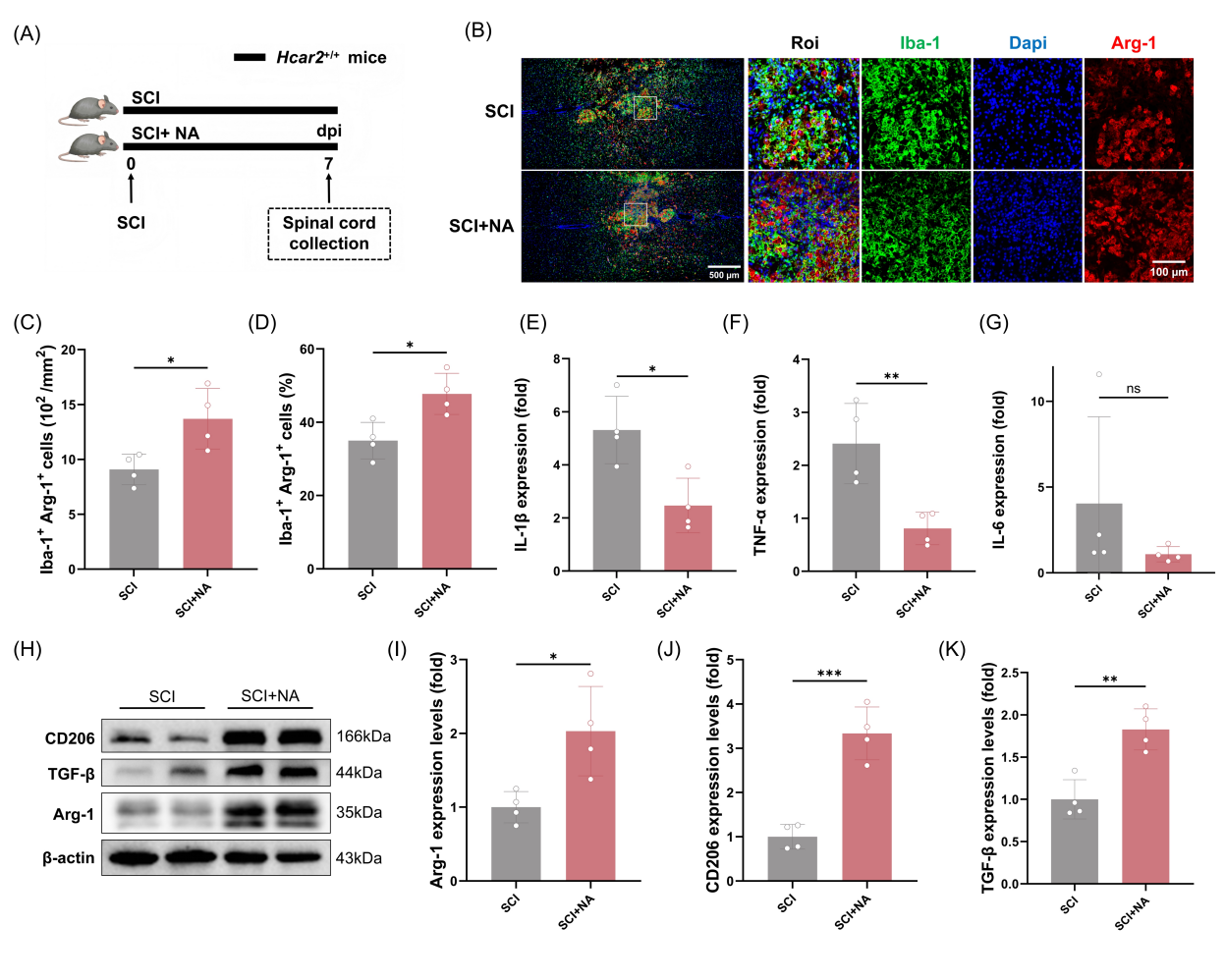


**Fig. S5. Niacin treatment promoted anti-inflammatory polarization and attenuated neuroinflammation in the injured spinal cord.** (A) Schematic of the experimental design for evaluating the anti-inflammatory effects of NA treatment in vivo at 7-dpi. (B) Representative immunofluorescence images of the lesion core at 7 dpi from SCI and SCI+NA groups. Sections were stained for Iba-1 (microglia, green), Arg-1 (M2 marker, red), and DAPI (nuclei, blue). Scale bars: 500 µm (overview), 100 µm (inset). (C, D) Quantification of Iba-1^+^Arg-1^+^ cell density (C) and the percentage of Arg-1^+^ microglia (D) (n = 4). (E–G) Multiplex flow cytometric analysis of IL-1β, TNF-α, and IL-6 in spinal cord lysates at 7 dpi: IL-1β (E), TNF-α (F), and IL-6 (G) (n = 4). (H) Representative Western blots of M2-like markers (Arg-1, CD206, TGF-β) in spinal cord lysates. (I–K) Densitometric quantification of Arg-1 (I), CD206 (J), and TGF-β (K) protein levels normalized to β-actin (n = 4). The data are presented as the means ± SD. Statistical significance was determined via Student’s t-test. **p <* 0.05, ***p <* 0.01, ****p <* 0.001; ns, not significant.
